# Supplementary material for: Structural coordination between active sites of a CRISPR reverse transcriptase-integrase complex
Source: Nat Commun. 2021 May 6;12:2571. doi: 10.1038/s41467-021-22900-y (PMC8102632; doi:10.1038/s41467-021-22900-y)
Supplement: Supplementary file 1 — Supplementary Information [file 41467_2021_22900_MOESM1_ESM.pdf]

Structural coordination between active sites of a CRISPR reverse transcriptase-integrase complex

Supplementary Information

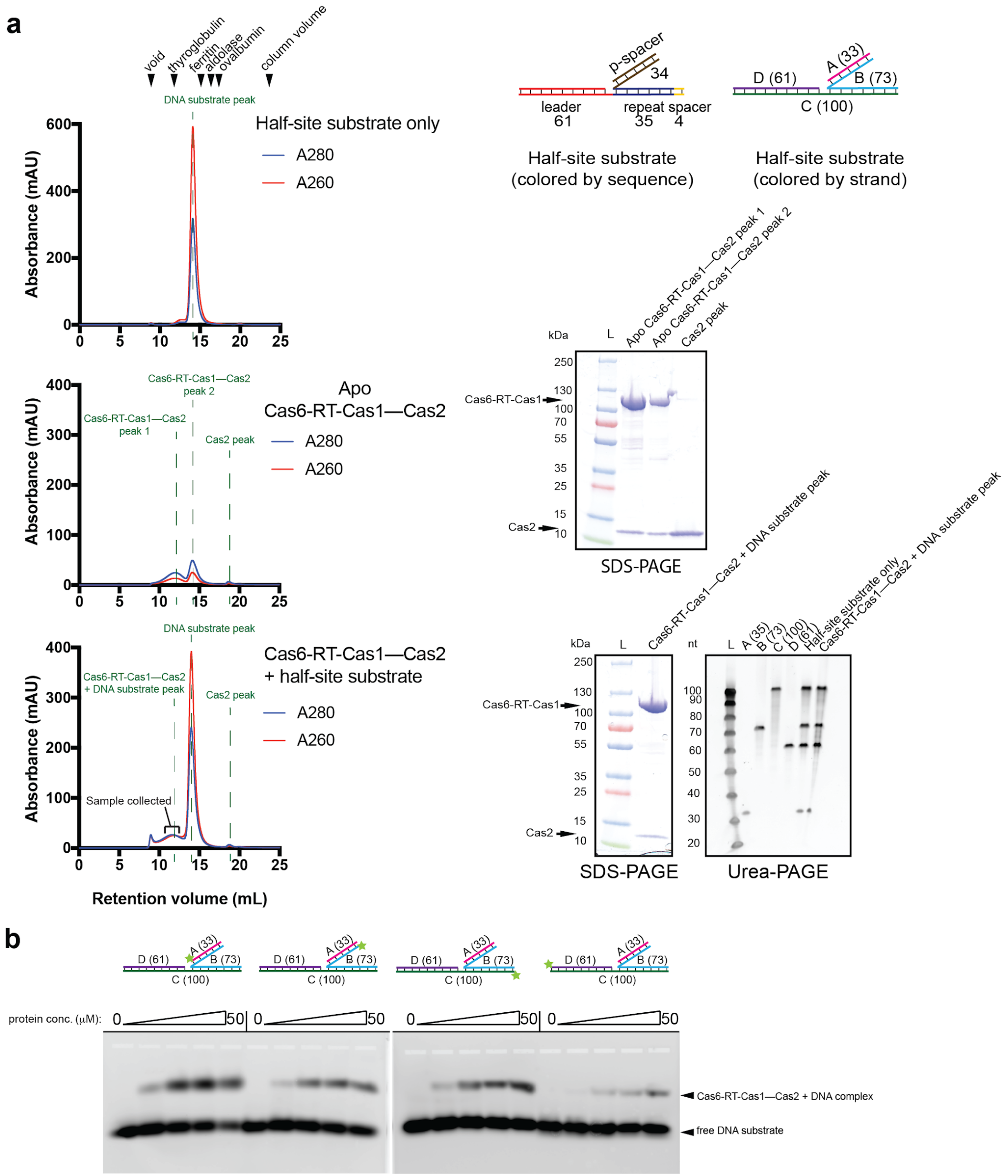

Supplementary Figure 1. **Complex formation. a** Gel filtration runs of DNA half-site substrate only, apo Cas6-RT-Cas1—Cas2, and Cas6-RT-Cas1—Cas2 + DNA half-site substrate. Cartoons of half-site substrate are shown colored by sequence (leader, red; repeat, blue; spacer, yellow; dsDNA protospacer, brown) and colored by strand with lengths of DNA strands shown (A, pink; B, light blue; C, green; D, purple). The absorbance at 280 nm (blue) and 260 nm (red) are shown and the void volume, column volume, and elution volumes of standard proteins are indicated by black arrowheads. The peaks representing the Cas6-RT-Cas1—Cas2 complexes, the sample used for cryo-EM data collection, and free DNA substrate are indicated by green dashed lines. SDS-PAGE gels and urea-PAGE gel of the elution samples are provided. Source data are provided as a Source Data file. **b** Electrophoretic mobility shift assays using fluorescent half-site substrates colored by strand with color-coding from **a**. Star indicates 6-carboxyfluorescein label. Results are representative of 3 independent experiments. Uncropped gels are available in a Source Data file.

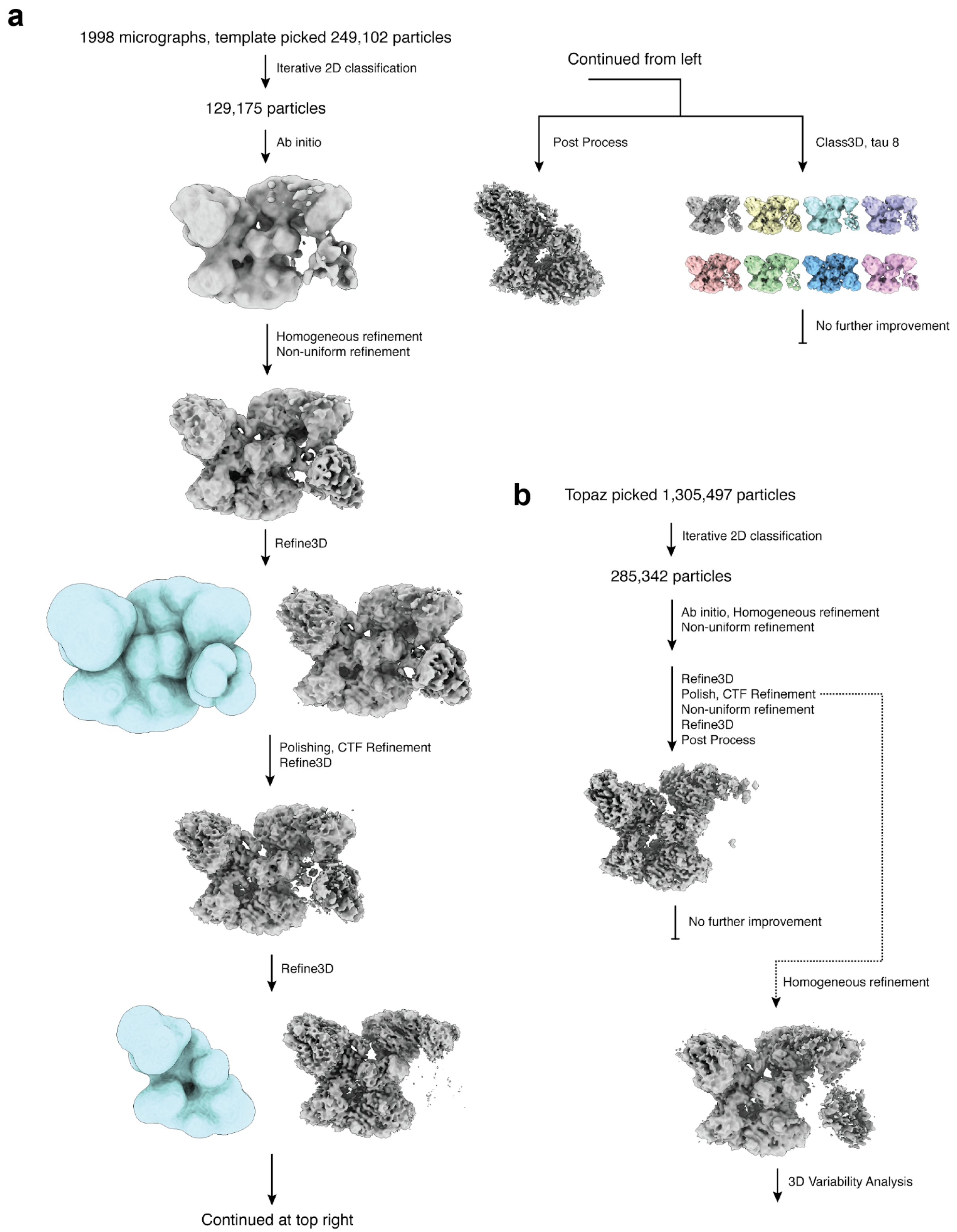

Supplementary Figure 2. **Cryo-EM processing pipeline. a**,**b** Cryo-EM data processing pipeline in RELION and cryoSPARC from RELION picked particles and Topaz picked particles respectively. Cryo-EM maps are colored gray, and masks used in RELION colored light blue. See Methods for details.

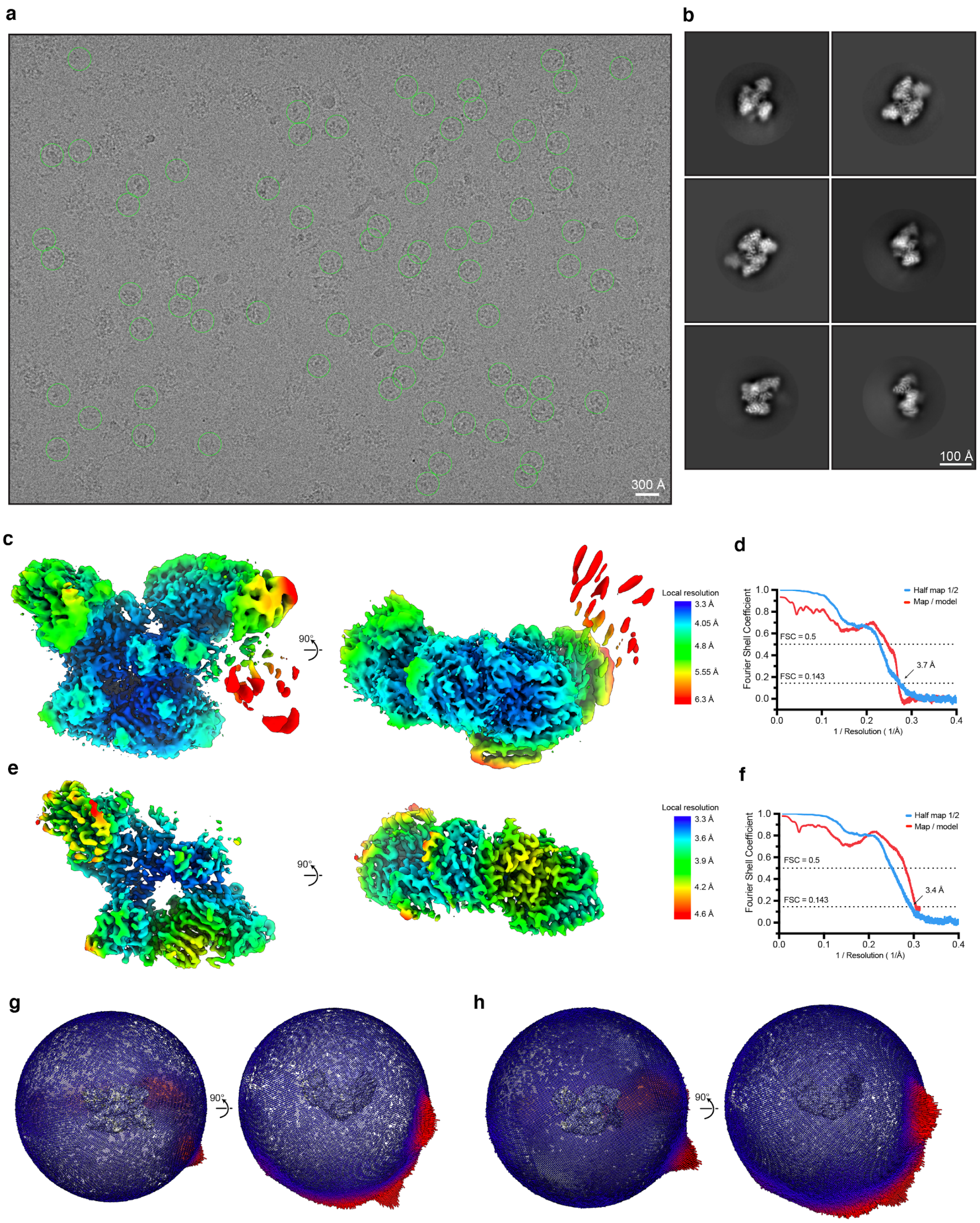

Supplementary Figure 3. **Cryo-EM validation. a** Representative micrograph with 10 Å lowpass filter, particles which contributed to the final reconstruction are circled. **b** Selected 2D class averages. Results for **a** and **b** are representative of 1996 micrographs (Supplementary Table 3). All micrographs are deposited in EMPIAR-10642 [https://dx.doi.org/10.6019/EMPIAR-10642]. **c**,**e** Local resolution estimated in RELION3.1 with map surfaces colored in rainbow as indicated, for full and partial complexes respectively. **d**,**f** Fourier Shell Correlation (FSC) for the two unfiltered half-maps (light blue) and final map versus model (red), for full and partial complexes respectively. Source data are provided as a Source Data file. **g**,**h** Angular distribution of particles with corresponding cryo-EM map, for full and partial complexes respectively.

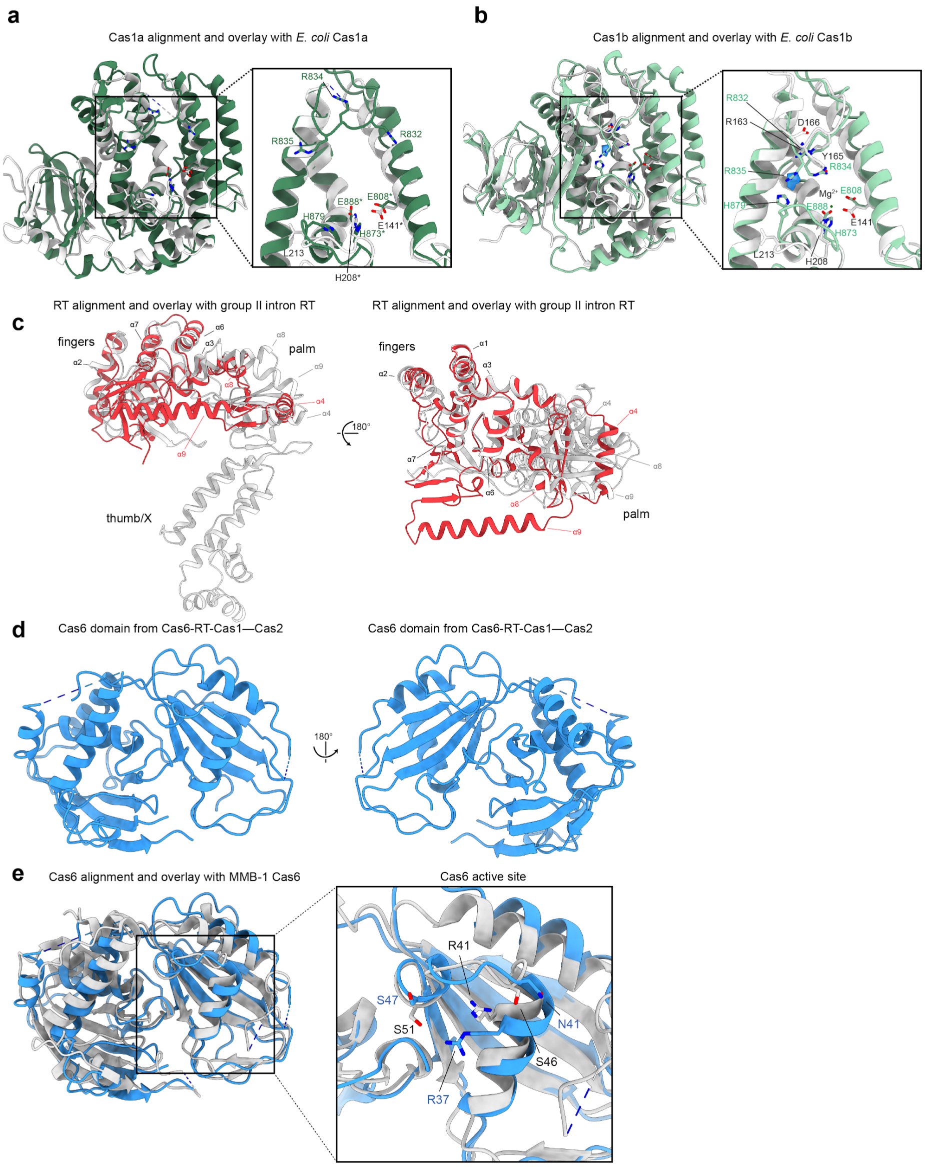

Supplementary Figure 4. **Architecture of Cas1, RT, and Cas6 domains.** **a** Alignment and overlay of Cas1a domain (dark green) from Cas6-RT-Cas1—Cas2 structure with *E. coli* Cas1a (white, PDB: 5DS5 [https://doi.org/10.2210/pdb5ds5/pdb]) and closeup of active site and surrounding residues, shown in stick configuration. Cas6-RT-Cas1—Cas2 residues are labeled in dark green, *E. coli* Cas1 residues labeled in black. Active site residues are labeled with an asterisk *. **b** Alignment and overlay of Cas1b domain (turquoise) with *E. coli* Cas1b (white, PDB: 5DS5 [https://doi.org/10.2210/pdb5ds5/pdb]) and closeup of active site and surrounding residues. **c** Alignment and overlay of RT domain (red) and group II intron RT (white, PDB: 6AR1 [https://doi.org/10.2210/pdb6AR1/pdb]) with helices from the finger and palm domains labeled. Closely-aligned helices labeled in black, offset helices labeled in red (Cas6-RT-Cas1—Cas2 RT domain) and gray (group II intron RT). **d** Architecture of Cas6 domain from Cas6-RT-Cas1—Cas2 structure and 90° rotation. **e** Alignment and overlay of Cas6 domain from Cas6-RT-Cas1—Cas2 structure (blue) with Cas6 domain from MMB-1 Cas6-RT-Cas1 fusion (PDB: 6DD5 [https://doi.org/10.2210/pdb6DD5/pdb], white) and closeup of active site residues, shown in stick configuration. Cas6-RT-Cas1—Cas2 residues are labeled in blue, MMB-1 Cas6 residues labeled in black.

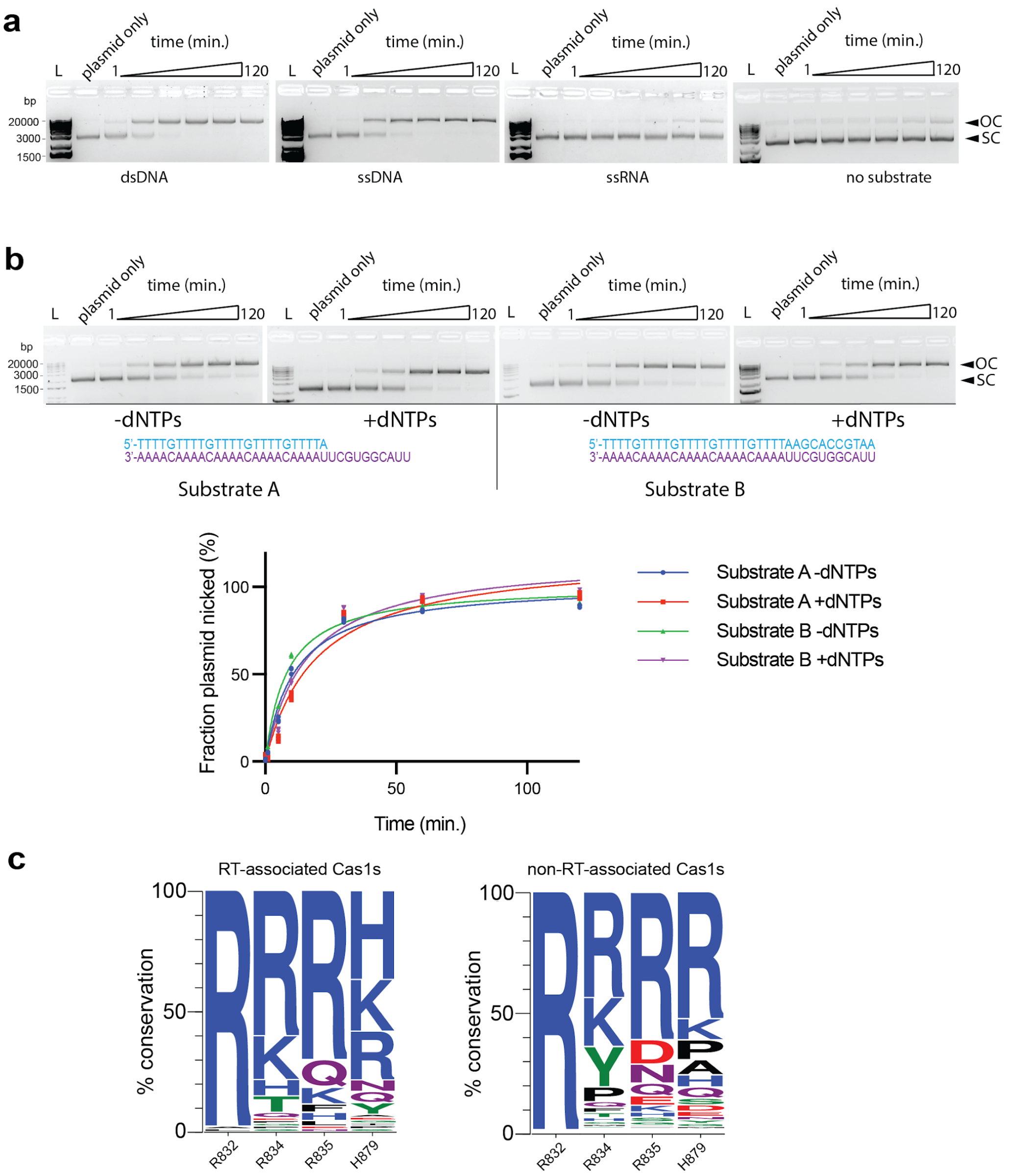

Supplementary Figure 5. **Cas6-RT-Cas1—Cas2 prefers DNA and DNA/RNA hybrid substrates over ssRNA substrates for cleavage-ligation. a** Time-course integration reactions with 35-nt dsDNA, ssDNA, ssRNA protospacers (4 μM) and a control with no protospacer. Supercoiled pCRISPR plasmid that functions as the target for integration (SC) and open-circle integration products (OC) are indicated. Results are representative of 3 independent experiments. **b** Time-course integration reactions comparing extent of plasmid-nicking with DNA/RNA hybrid substrates (4 μM) with and without dNTPs and quantification. Substrate sequences are shown (DNA, blue; RNA, purple). Fraction plasmid nicked is calculated as the fraction of open circular products relative to all plasmid (n = 3 biologically independent experiments). Experimental fits are shown as solid lines. Statistical significance of fraction plasmid nicked after two hours was assessed by comparing results to the results with ssRNA from Fig. 2c using unpaired, two-tailed T tests (α = 0.05) (P = 0.0002, Substrate A - dNTPs; P = 0.0001, Substrate A + dNTPs; P = 0.0002, Substrate B - dNTPs; P = 0.0001, Substrate B + dNTPs). Source data are provided as a Source Data file. **c** Sequence logos indicating the most conserved residues of the R832, R834, R835, and H879 motif in RT-associated Cas1s (left) and non-RT-associated Cas1s (right) following sequence alignment across a diverse selection of Cas1s. Uncropped gels are available in a Source Data file.

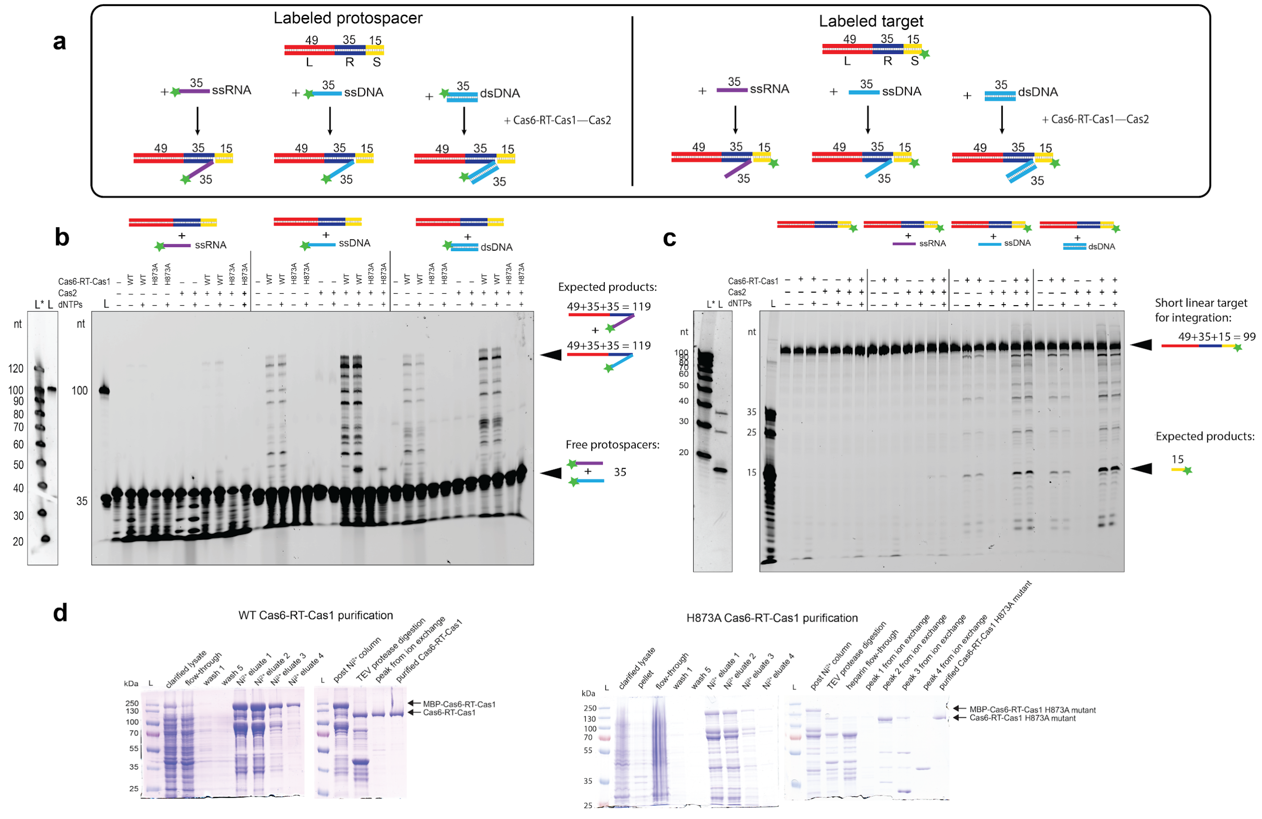

Supplementary Figure 6. **Cas6-RT-Cas1—Cas2 catalyzes ligation of DNA and RNA protospacers into the CRISPR array. a** Schematic of *in vitro* integration reaction of DNA or RNA protospacer into a short linear dsDNA containing the CRISPR repeat as the target for integration. The lengths of the leader (L, red), repeat (R, dark blue), spacer (S, yellow), and protospacers (DNA, light blue; RNA, purple) are indicated. Star indicates 6-carboxyfluorescein label. **b** Cleavage-ligation assays conducted with fluorescent dsDNA, ssDNA, and ssRNA protospacers (1 µM) and unlabeled short linear target containing the CRISPR repeat. Expected products and their lengths are indicated. On the left, a non-fluorescent ladder L* is shown next to the fluorescent ladder L, visualized after SYBR Gold poststaining. **c** Cleavage-ligation assays conducted with fluorescent target (bottom strand labeled). Expected products and their lengths are indicated. Results for **b** and **c** are representative of 3 independent experiments. **d** SDS-PAGE gels of WT Cas6-RT-Cas1 and H873A Cas6-RT-Cas1 mutant purifications, representative of 1 independent experiment. Uncropped gels are available in a Source Data File.

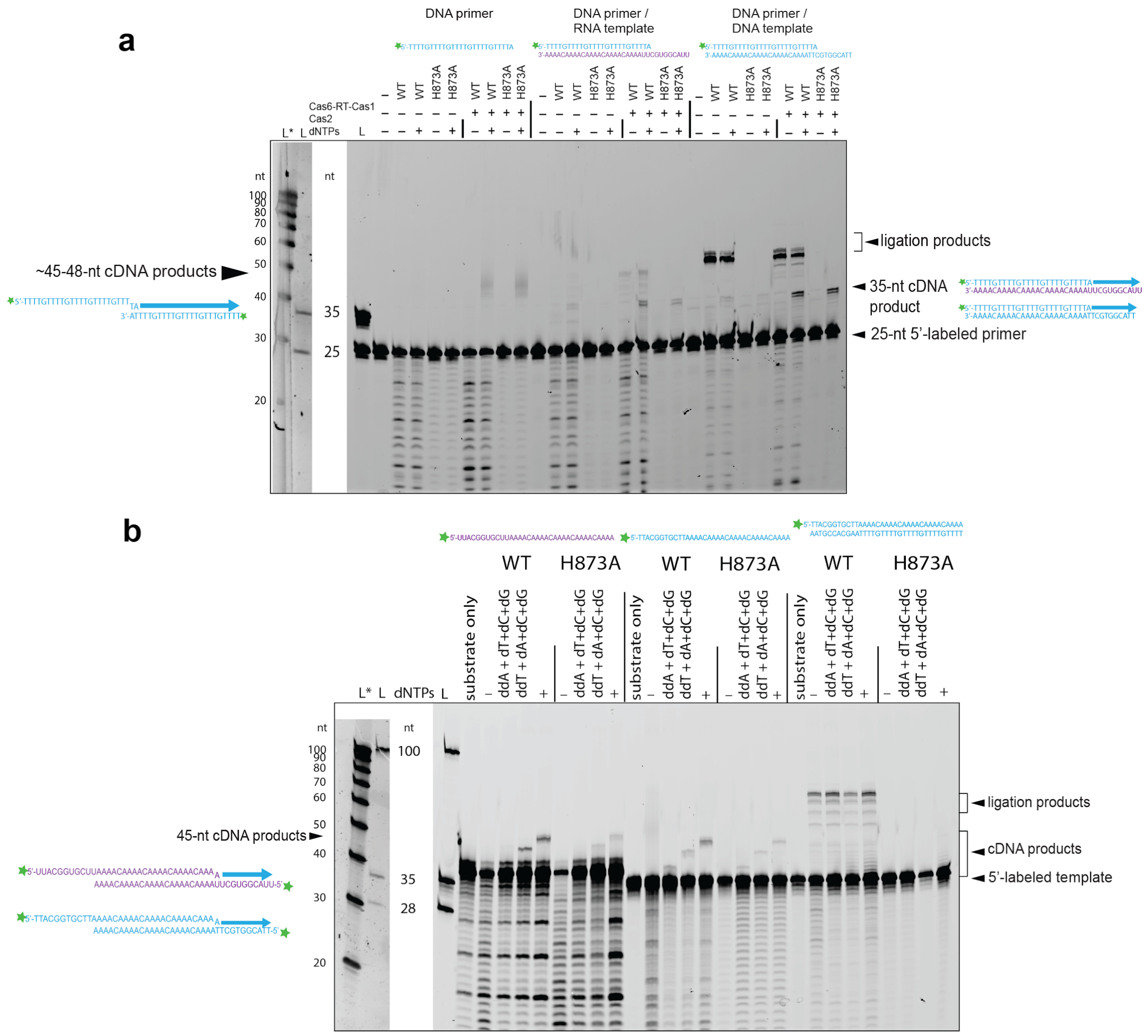

Supplementary Figure 7. **Cas6-RT-Cas1 catalyzes cDNA synthesis with minimal primer-template homology.** **a** Template-driven cDNA synthesis reactions off a fluorescent DNA primer annealed to DNA and RNA templates with WT and H873A Cas6-RT-Cas1 complexed with Cas2. Substrate sequences are shown (DNA, blue; RNA, purple) and arrows indicate expected cDNA synthesis reactions. Star indicates 6-carboxyfluorescein label. On the left, a non-fluorescent ladder L* is shown next to the fluorescent ladder L, visualized after SYBR Gold poststaining. **b** Template-driven cDNA synthesis reactions in the absence of different dNTPs and in the presence of added ddNTPs with DNA or RNA template with no pre-annealed primer. Arrows indicate expected cDNA synthesis reactions. Uncropped gels are available in a Source Data file. Results for **a** and **b** are representative of 3 independent experiments.

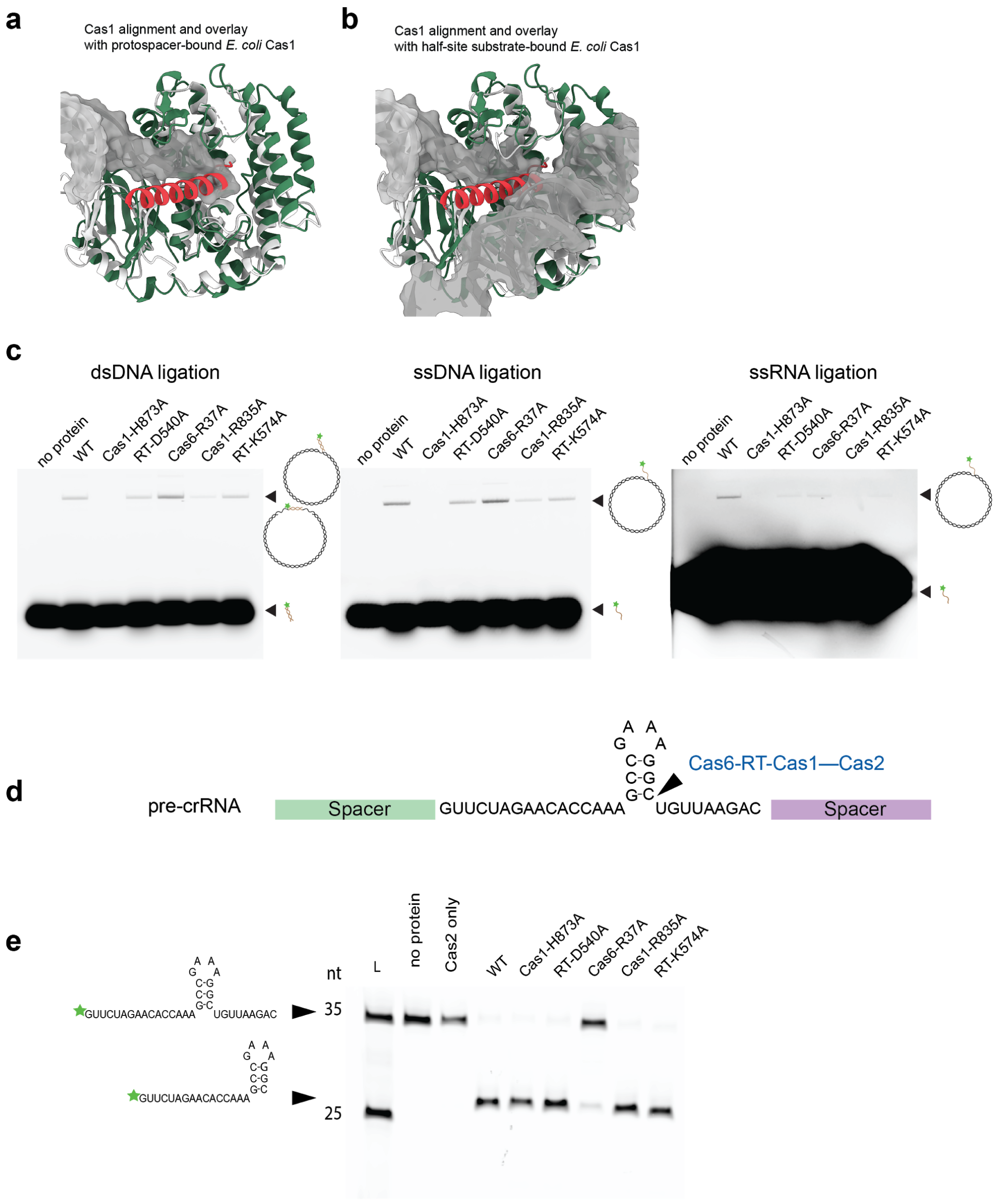

Supplementary Figure 8. **Crosstalk between Cas1, RT, and Cas6 domains. a** Alignment and overlay of Cas1a domain (dark green) with Cas1a from protospacer-bound *E. coli* Cas1—Cas2 structure (white, PDB: 5DS5 [https://doi.org/10.2210/pdb5ds5/pdb]). Protospacer substrate shown in gray surface representation. RT-helix is colored red. **b** Alignment and overlay of Cas1a domain (dark green) with Cas1a from half-site substrate-bound *E. coli* Cas1—Cas2 structure (white, PDB: 5VVJ [https://doi.org/10.2210/pdb5VVJ/pdb]). Half-site substrate shown in gray surface representation. **c** Ligation of fluorescent 35-nt dsDNA (0.5 μM), ssDNA (0.5 μM), and ssRNA (4 μM) protospacer into target pCRISPR by WT and mutant Cas6-RT-Cas1—Cas2s. Integration products and free protospacer are indicated and schematized. Star indicates 6-carboxyfluorescein label. **d** Sequence and predicted secondary structure of repeat with predicted cleavage site indicated. **e** crRNA processing activity assay comparing WT and mutant Cas6-RT-Cas1—Cas2s. Sequence and predicted secondary structure of CRISPR repeat RNA and the predicted product are indicated. Star indicates 6-carboxyfluorescein label. Results for **c** and **e** are representative of 3 independent experiments. Uncropped gels are available in a Source Data file.

Protein	Sequence
Cas6-RT-Cas1	MILPSFPDLTGLVVNLKFTARAEFSLNHEMAVDAFLRHSLNLGESYSHHLS<br>IITPENGRLFYREGDTYRFVVIAMGNQQQTNSIWHTLINHLRKLPDSAPITD<br>KQAPLRNNIKLESLNDLFDGIPVSSKESLDAYTLQRAMEQGLAWHKAANL<br>TEQPLDIQWYWQSTVRILHADHKQHKGEQRYCRDAVQLTPLLLLKRIYET<br>LNNVATYFGLKTNKNTTENHQAWLKEQAQYIEIQHPDLYWIDTPYFGKDA<br>EKNTLGGMAGNFTLSLKPGIEPGLLAMLILTQMVGVGQRRTSGLGKYWL<br>KHSLKHAHLILGLKPNRVTRSQTLLDCIIQPHIISQAIAEIEKKTNIDTLNERT<br>LSQVQSAIGQLRKHQYQAPKLQGFTIPKKDGTERLLAVSPLYDRILQKAAA<br>IVLTPGLDAIMSQASYGYRKGLSRQQVRYEIQNAYRQGYHWVYESDIEDF<br>FDAVYRPQLINRLKSLLGNDPLWEQIESWLGQDIHIKDTIIERTPNLGLPQG<br>SPLSPLLANFILDDFDSDLETHGFKIIRFADDFIILCKSQHEAQQAAHAVEQ<br>SLKEVKLSINVEKTHIIQLNQGFRFLGYLFREDHAIEIAGEKSDGRTTFAAE<br>QTPTNLPPWLANLGTKSPQPLADDDLPKKSYGQIETQGTHLVLAGDAQIIT<br>TDNQNLIVKKDDKITHKISLEQLHAVTLIGLHTMTLPAKHRLLEHKIPVHIAD<br>RTGRYLGAVTSFQPAQNNYKNWFIQLQMCDREPFAHAIAQQIVISRIHNQ<br>RQTLLKRKAHRKQLQQTLSNLKKLQYKVTAATKRSSLNGLEGSATREYFQ<br>QFNLFLPEWAHFSKRTRRPPKDPFNVLLSLGYTILYSHTDAILQSAGFITW<br>KGIYHQQSAAHAALASDIMESYRHLVERYAIYIINHGQIKQDDFRQEKDHL<br>GQDTIRLSAEARRRYVGGLINRFQKFSKDKTLHQHLYQQAQQLKNAMHN<br>QQSSQFQVWKELK*
Cas2	MKHYLICFDVQHDKTRAKLSRLLEKYGPRVQGSVFEVSFKTPDRKRQLE<br>YKIHQIIKQSNTEENNIRFYNLNKDTIKHSHDINGNPIAQLPAAIVL*

Supplementary Table 1. Sequences of type III *Thiomicrospira* Cas6-RT-Cas1 and Cas2 proteins.

Name/Description	Sequence	Figure
Half-site substrate protospacer-repeat-spacer	GTAAAATCGAAGGAATGCTAAGGAAGCTAAAATGGTTCTAG AACACCAAAGCCGAAAGGCTGTTAAGACAGCT	1, S1
Half-site substrate protospacer	ATTTTAGCTTCCTTAGCATTCCTTCGATTTTAC	1, S1
Half-site substrate spacer-repeat-leader	AGCTGTCTTAACAGCCTTTCGGCTTTGGTGTTCTAGAACTC CGGTTGAAATTCTATCTGCTTATTCAATGAGTTGCAATGCAT TTTCCGCACCAGAATGA	1, S1
Half-site substrate leader	TCATTCTGGTGCGGAAAATGCATTGCAACTCATTGAATAAG CAGATAGAATTTCAACCGGA	1, S1
Fluorescent half-site substrate protospacer-repeat-spacer	/56-FAM/GTAAAATCGAAGGAATGCTAAGGAAGCTAAAATGG TTCTAGAACACCAAAGCCGAAAGGCTGTTAAGACAGCT	S1
Fluorescent half-site substrate protospacer	/56-FAM/ATTTTAGCTTCCTTAGCATTCCTTCGATTTTAC	S1
Fluorescent half-site substrate spacer-repeat-leader	/56-FAM/AGCTGTCTTAACAGCCTTTCGGCTTTGGTGTTCTA GAACTCCGGTTGAAATTCTATCTGCTTATTCAATGAGTTGC AATGCATTTTCCGCACCAGAATGA	S1
Fluorescent half-site substrate leader	/56-FAM/TCATTCTGGTGCGGAAAATGCATTGCAACTCATTG AATAAGCAGATAGAATTTCAACCGGA	S1
Fluorescent 35-mer ssDNA protospacer and (+) strand for dsDNA protospacer and DNA template used for cDNA synthesis reactions	/56-FAM/TTACGGTGCTTAAAACAAAACAAAACAAAACAAAA	2, 6, S6, S7, S8
RC for fluorescent and non-fluorescent dsDNA protospacer	TTTTGTTTTGTTTTGTTTTGTTTTAAGCACCGTAA	2, 6, S5, S6, S7, S8
Fluorescent 35-mer RNA protospacer and RNA template used for cDNA synthesis reactions	/56-FAM/UUACGGUGCUUAAAACAAAACAAAACAAAACAAA A	2, 6, S6, S7, S8
35-mer ssDNA protospacer and (+) strand for dsDNA protospacer and DNA template used for cDNA synthesis reactions	TTACGGTGCTTAAAACAAAACAAAACAAAACAAAA	2, 5, S5, S6, S7
35-mer RNA protospacer and RNA template used for cDNA synthesis reactions	UUACGGUGCUUAAAACAAAACAAAACAAAACAAAA	2, 5, S5, S6, S7
25-mer DNA strand for RNA/DNA hybrid	TTTTGTTTTGTTTTGTTTTGTTTTA	S5
15-mer ssDNA protospacer and (+) strand for dsDNA	AGCTGTTGATAAGCA	3
RC for dsDNA protospacer	TGCTTATCAACAGCT	3

35-mer ssDNA protospacer and (+) strand for dsDNA	AGCTGTTAAGTACCCTTGGATCAGGCGGATAAGCA	3
RC for dsDNA protospacer	TGCTTATCCGCCTGATCCAAGGGTACTTAACAGCT	3
55-mer ssDNA protospacer and (+) strand for dsDNA	AGCTGTTAAGTACCAATGTTCCAGACCATCCCTTCTTGGATCAGGCGGATAAGCA	3
RC for dsDNA protospacer	TGCTTATCCGCCTGATCCAAGAAGGGATGGTCTGGAACATTGGTACTTAACAGCT	3
75-mer ssDNA protospacer and (+) strand for dsDNA	AGCTGTTAAGTACCAATGTTCCAGACCATCCCTTCAAGTCTTTGTACTGCGTCCATTGGATCAGGCGGATAAGCA	3
RC for dsDNA protospacer	TGCTTATCCGCCTGATCCAATGGACGCAGTACAAAGACTTGAAGGGATGGTCTGGAACATTGGTACTTAACAGCT	3
95-mer ssDNA protospacer and (+) strand for dsDNA	AGCTGTTAAGTACCAATGTTCCAGACCATCCCAGTTAACGCATCCATAGCAATTCAAGTCTTTGTACTGCGTCCATTGGATCAGGCGGATAAGCA	3
RC for dsDNA protospacer	TGCTTATCCGCCTGATCCAATGGACGCAGTACAAAGACTTGAATTGCTATGGATGCGTTAACTGGGATGGTCTGGAACATTGGTACTTAACAGCT	3
115-mer ssDNA protospacer and (+) strand for dsDNA	AGCTGTTAAGTACCAATGTCACGTACCATTCCAGACCATCCCAGTTAACGCTTACGATTCAATCCATAGCAATTCAAGTCTTTGTACTGCGTCCATTGGATCAGGCGGATAAGCA	3
RC for dsDNA protospacer	TGCTTATCCGCCTGATCCAATGGACGCAGTACAAAGACTTGAATTGCTATGGATTGAATCGTAAGCGTTAACTGGGATGGTCTGGAATGGTACGTGACATTGGTACTTAACAGCT	3
Full-site assay protospacer	GTAAAATCGAAGGAATGCTAAGGAAGCTAAAATG	4
RC	CATTTTAGCTTCCTTAGCATTCCTTCGATTTTAC	4
Fluorescent 25-mer DNA primer used for cDNA synthesis reactions	/56-FAM/TTTTGTTTTGTTTTGTTTTGTTTTA	5, S7
Fluorescent RNA repeat	/56-FAM/GUUCUAGAACACCAAAGCCGAAAGGCUGUUAAGAC	6, S8
Linear DNA target (+) strand	TTGGTCTTAACAGCTGTCTTAACAGCCTTTCGGCTTTGGTGTTCTAGAACTCCGGTTGAAATTCTATCTGCTTATTCAATGAGTTGCAATGCATTTTCC/3ddC/	S6
RC for fluorescent and non-fluorescent linear DNA target	GGAAAATGCATTGCAACTCATTGAATAAGCAGATAGAATTTCAACCGGAGTTCTAGAACACCAAAGCCGAAAGGCTGTTAAGACAGCTGTTAAGACCAA/3ddC/	S6
Fluorescent linear DNA target (+) strand	/56-FAM/TTGGTCTTAACAGCTGTCTTAACAGCCTTTCGGCTTTGGTGTTCTAGAACTCCGGTTGAAATTCTATCTGCTTATTCAATGAGTTGCAATGCATTTTCC/3ddC/	S6
pCRISPR_full-site cloning primer 1	AAAGCCGAAAGGCTGTTAAGACAGAGAAAAAAATCACTGGATATACCACCGTTG	

pCRISPR_full-site cloning primer 2	CACCATATTTTAATGTTGATTTAGCTCCTGAAAATCTCGATAACTCAAAAAATACG	
pCRISPR_full-site cloning primer 3	TTTTTGAGTTATCGAGATTTTCAGGAGCTAAATCAACATTAAAATATGGTGCGGGAACT	
pCRISPR_full-site cloning primer 4	AACGGTGGTATATCCAGTGATTTTTTTCTCTGTCTTAACAGCCTTTCGGCTTT	
pCRISPR cloning primer 1	TAACGCGGCTGTCTTGTATAACAATTTCACACAGGAAACAGCTATGACC	
pCRISPR cloning primer 2	GATAACTTTGTGGCGTTTTGGTGCCGCTGTGCGGTTTGCGTATTGGGC	
pCRISPR cloning primer 3	AAGCGGAAGAGCGCCCAATACGCAAACCGCACAGCGGCACCAAAACG	
pCRISPR cloning primer 4	AGCCTTTCGGCTTTGGTGTTCTAGAACAGTACAAAGACTTGAAGGGATG	
pCRISPR cloning primer 5	AGTCTTTGTACTGTTCTAGAACACCAAAGCCGAAAGGCTGTTAAGACTC	
pCRISPR cloning primer 6	TCATAGCTGTTTCCTGTGTGAAATTGTTATACAAGACAGCCGCGTTAGC	
pCas6-RT-Cas1_expression cloning primer 1	TTCAAGTATGGAAGGAGTTGAAGTGAATTGGAAGTGGATAACGGATCCG	
pCas6-RT-Cas1_expression cloning primer 2	GGGAAAGACGGAAGAATCATAGCATTGGATTGGAAGTACAGGTTTTCCT	
pCas6-RT-Cas1_expression cloning primer 3	GAAAACCTGTACTTCCAATCCAATGCTATGATTCTTCCGTCTTTCCCCG	
pCas6-RT-Cas1_expression cloning primer 4	GATCCGTTATCCACTTCCAATTCACTTCAACTCCTTCCATACTTGAAAC	
pCas2_expression cloning primer 1	AATTACCTGCTGCAATCGTTCTATAAATTGGAAGTGGATAACGGATCCG	
pCas2_expression cloning primer 2	CAAATCAGGTAGTGTTTCATAGCATTGGATTGGAAGTACAGGTTTTCCT	
pCas2_expression cloning primer 3	TGTACTTCCAATCCAATGCTATGAAACACTACCTGATTTGCTTTGATGT	
pCas2_expression cloning primer 4	ATCCGTTATCCACTTCCAATTTATAGAACGATTGCAGCAGGTAATTGAG	
pCas6-RT-Cas1H873A_expression cloning primer 1	GCACAGCAGTCGGCCGC	
pCas6-RT-Cas1H873A_expression cloning primer 2	ATAGATGCCTTTCCATGTGATAAACCC	
pCas6-RT-Cas1D540A_expression cloning primer 1	GCAGATTTCATTATTTTATGTAAGTCACAACATGAAGCCC	

pCas6-RT-Cas1D540A_expression cloning primer 2	AGCAAAACGGATGATCTTGAACCC	
pCas6-RT-Cas1R37A_expression cloning primer 1	GCACACTCTTTAAATTTGGGCGAGAGCTA	
pCas6-RT-Cas1R37A_expression cloning primer 2	TAAGAATGCATCCACAGCCATTTCG	
pCas6-RT-Cas1K574A_expression cloning primer 1	GCAACGCACATCATTCAGCTGAAC	
pCas6-RT-Cas1K574A_expression cloning primer 2	GCAACGCACATCATTCAGCTGAAC	
pCas6-RT-Cas1R835A_expression cloning primer 1	GCACCTCCTAAAGATCCTTTCAATGTGCTG	
pCas6-RT-Cas1R835A_expression cloning primer 2	ACGTGTGCGCTTCGAAAAATG	

Supplementary Table 2. DNA and RNA oligonucleotides used in this study. RC indicates the complementary strand of the previous oligonucleotide. Sequences are written 5’ to 3’.

	Cas6-RT-Cas1—Cas2 (masked partial complex)<br>(EMD-22855 [https://www.ebi.ac.uk/pdbe/entry/emdb/EMD-22855])<br>(PDB 7KFT [https://doi.org/10.2210/pdb7KFT/pdb])	Cas6-RT-Cas1—Cas2 (full complex)<br><br>(EMD-22856 [https://www.ebi.ac.uk/pdbe/entry/emdb/EMD-22856])<br>(PDB 7KFU [https://doi.org/10.2210/pdb7KFU/pdb])
**Data collection and processing**		
Magnification	64,000x	64,000x
Voltage (kV)	300	300
Electron exposure (e–/Å^2)	49.98	49.98
Defocus range (μm)	1.0 - 3.0	1.0 - 3.0
Pixel size (Å)	1.187	1.187
Symmetry imposed	C1	C1
Initial particle images (no.)	249,102	249,102
Final particle images (no.)	129,175	129,175
Map resolution (Å)	3.4	3.7
FSC threshold	0.143	0.143
Map resolution range (Å)	3.35-5.68	3.39-9.07
**Refinement**		
Initial model used (PDB code)	7KFT	7KFU
Model resolution (Å)	3.3 / 3.5	3.7 / 4.0
FSC threshold	FSC = 0.143 / FSC = 0.5	FSC = 0.143 / FSC = 0.5
Map sharpening *B* factor (Å^2)	-111.2	-66.03
Model composition		
Non-hydrogen atoms	11680	21848
Protein residues	1427	2674
Ligands	0	0
B factors (Å^2)		
Protein	57.06	183.8
R.m.s. deviations		
Bond lengths (Å)	0.005	0.006
Bond angles (°)	0.824	0.821
Validation		
MolProbity score	1.63	1.68
Clashscore	5.54	6.27
Ramachandran plot		
Favored (%)	95.24	95.15
Allowed (%)	4.76	4.85
Disallowed (%)	0	0

Supplementary Table 3. Cryo-EM data collection, processing, and refinement**.**
