# Supplementary material for: Structural coordination between active sites of a CRISPR reverse transcriptase-integrase complex
Source: Nat Commun. 2021 May 6;12:2571. doi: 10.1038/s41467-021-22900-y (PMC8102632; doi:10.1038/s41467-021-22900-y)
Supplement: Supplementary file 5 — Description of Additional Supplementary Files [file 41467_2021_22900_MOESM5_ESM.docx]

Description of additional supplementary information

Title: Supplementary Movie 1.

Description: 3D variability analysis of cryo-EM data heterogeneity in Cas6-RTCas1-Cas2 complex.

Title: Supplementary Data 1.

Description: CRISPR locus of Thiomicrospira system from groundwater.
