# Supplementary material for: Structural coordination between active sites of a CRISPR reverse transcriptase-integrase complex
Source: Nat Commun. 2021 May 6;12:2571. doi: 10.1038/s41467-021-22900-y (PMC8102632; doi:10.1038/s41467-021-22900-y)
Supplement: Supplementary file 6 — Reporting Summary [file 41467_2021_22900_MOESM6_ESM.pdf]

Corresponding author(s): Jennifer A. Doudna

Last updated by author(s): Mar 25, 2021

Reporting Summary

Nature Research wishes to improve the reproducibility of the work that we publish. This form provides structure for consistency and transparency in reporting. For further information on Nature Research policies, see our Editorial Policies and the Editorial Policy Checklist.

Statistics

For all statistical analyses, confirm that the following items are present in the figure legend, table legend, main text, or Methods section.

n/a	Confirmed	
☐	☒	The exact sample size (*n*) for each experimental group/condition, given as a discrete number and unit of measurement
☐	☒	A statement on whether measurements were taken from distinct samples or whether the same sample was measured repeatedly
☐	☒	The statistical test(s) used AND whether they are one- or two-sided<br>*Only common tests should be described solely by name; describe more complex techniques in the Methods section.*
☒	☐	A description of all covariates tested
☒	☐	A description of any assumptions or corrections, such as tests of normality and adjustment for multiple comparisons
☐	☒	A full description of the statistical parameters including central tendency (e.g. means) or other basic estimates (e.g. regression coefficient) AND variation (e.g. standard deviation) or associated estimates of uncertainty (e.g. confidence intervals)
☐	☒	For null hypothesis testing, the test statistic (e.g. *F*, *t*, *r*) with confidence intervals, effect sizes, degrees of freedom and *P* value noted<br>*Give P values as exact values whenever suitable.*
☒	☐	For Bayesian analysis, information on the choice of priors and Markov chain Monte Carlo settings
☒	☐	For hierarchical and complex designs, identification of the appropriate level for tests and full reporting of outcomes
☒	☐	Estimates of effect sizes (e.g. Cohen's *d*, Pearson's *r*), indicating how they were calculated

Our web collection on statistics for biologists contains articles on many of the points above.

Software and code

Policy information about availability of computer code

Data collection	Amersham Typhoon FLA (GE Healthcare), ChemiDoc MP (BioRad), Fluorescent plate reader (Biotek), SerialEM 3.6
Data analysis	ImageQuant TL 8.2 (GE Healthcare), Image Lab 6.0 (BioRad), Prism 7 version 7.0c (GraphPad), RELION 3.0-3.1, MotionCor2, CTFFIND 4.1, cryoSPARCv2, UCSF pyem v0.5, Phenix 1.18, Coot 0.8.9.2-0.9, Molprobity 4.5, Chimera 1.14, ChimeraX 0.93-1.0, Topaz 0.2.3, GNU Image Manipulation Program, Adobe Illustrator 2020, MAFFT v7.407 LINSI, Bowtie2 v.2.3.4.15, SnapGene 5.0.8

For manuscripts utilizing custom algorithms or software that are central to the research but not yet described in published literature, software must be made available to editors and reviewers. We strongly encourage code deposition in a community repository (e.g. GitHub). See the Nature Research guidelines for submitting code & software for further information.

Data

Policy information about availability of data

All manuscripts must include a data availability statement. This statement should provide the following information, where applicable:

- Accession codes, unique identifiers, or web links for publicly available datasets
- A list of figures that have associated raw data
- A description of any restrictions on data availability

The atomic model of the partial Cas6-RT-Cas1—Cas2 complex masked for the stable density is in the Protein Data Bank (PDB) under 7KFT [https://doi.org/10.2210/pdb7KFT/pdb] and the corresponding map is deposited in the Electron Microscopy Data Bank (EMDB) under EMD-22855 [https://www.ebi.ac.uk/pdbe/entry/emdb/EMD-22855]. The atomic model of the full Cas6-RT-Cas1—Cas2 complex is in the Protein Data Bank (PDB) under 7KFU [https://doi.org/10.2210/pdb7KFU/pdb] and the corresponding map is deposited in the Electron Microscopy Data Bank (EMDB) under EMD-22856 [https://www.ebi.ac.uk/pdbe/entry/emdb/EMD-22856]. The original micrograph movies and final particle stack are deposited in the Electron Microscopy Public Image Archive (EMPIAR) under EMPIAR-10642 [https://

dx.doi.org/10.6019/EMPIAR-10642]. The uncropped images for the main text or supplementary figures and source data from Fig. 2-6 and Supplementary Fig. 1, 3, 5-8 are provided with this paper as a Source Data file. All relevant data are available from the authors.

Field-specific reporting

Please select the one below that is the best fit for your research. If you are not sure, read the appropriate sections before making your selection.

☒ Life sciences ☐ Behavioural & social sciences ☐ Ecological, evolutionary & environmental sciences

For a reference copy of the document with all sections, see nature.com/documents/nr-reporting-summary-flat.pdf

Life sciences study design

All studies must disclose on these points even when the disclosure is negative.

Sample size	Sample size is indicated in the figure legends. For all biochemistry assays, experiments were performed in triplicates with representative results shown. A sample size of 3 independent biological replicates was chosen for experiments, as is standard for similar biochemistry experiments, following resource constraints. This sample size is sufficient to show statistical significance assessed using unpaired, two-tailed T tests (α = 0.05).
Data exclusions	No data was excluded from the analyses.
Replication	Replicates indicate independent samples carried out as experimental replicates, n=3. All attempts at replication were successful.
Randomization	Randomization is not relevant to this study, because the protein/DNA/RNA samples allocated into experimental groups were drawn from the same stocks.
Blinding	Blinding is not relevant to this study, because the protein/DNA/RNA samples allocated into experimental groups were drawn from the same stocks.

Reporting for specific materials, systems and methods

We require information from authors about some types of materials, experimental systems and methods used in many studies. Here, indicate whether each material, system or method listed is relevant to your study. If you are not sure if a list item applies to your research, read the appropriate section before selecting a response.

Materials & experimental systems

n/a	Involved in the study	
☒	☐	Antibodies
☒	☐	Eukaryotic cell lines
☒	☐	Palaeontology and archaeology
☒	☐	Animals and other organisms
☒	☐	Human research participants
☒	☐	Clinical data
☒	☐	Dual use research of concern

Methods

n/a	Involved in the study	
☒	☐	ChIP-seq
☒	☐	Flow cytometry
☒	☐	MRI-based neuroimaging
