# Supplementary material for: Structural coordination between active sites of a CRISPR reverse transcriptase-integrase complex
Source: Nat Commun. 2021 May 6;12:2571. doi: 10.1038/s41467-021-22900-y (PMC8102632; doi:10.1038/s41467-021-22900-y)
Supplement: Supplementary file 2 — Peer Review File [file 41467_2021_22900_MOESM2_ESM.pdf]

REVIEWER COMMENTS

Reviewer #1 (Remarks to the Author):

Wang et al. present structural and functional studies of an unusual CRISPR-Cas integration complex consisting of Cas2 and a Cas1 protein, which is naturally linked to reverse transcriptase and Cas6 enzymes. The structure reveals an unusual higher-order structure in which Cas1 and Cas2 interact in a non-canonical way and the active sites of two (out of four) Cas1 subunits are blocked by a helix from the RT enzyme. Functional studies reveal that while the enzyme can ligate both ssDNA, dsDNA, and ssRNA substrates, only dsDNA can get fully integrated in vitro and in vivo.

This manuscript presents novel and highly interesting insights into an unusual CRISPR-Cas system, which strongly complements and extends earlier studies of RT-containing integration systems. The structural work is sound and the conclusions drawn from this are mostly warranted given the modest resolution (3.7 Å). The text is illustrated by beautiful figures that are mostly very clear. Nevertheless, I find that some of the biochemistry and functional studies require additional support to complete the story.

A major outstanding question is how the RT domain works in connection with the integration functions as classically supported by Cas1/Cas2. The functional studies address this in several ways, initially through analysis of ligation of ssDNA, ssRNA, and dsDNA into a plasmid-borne CRISPR array. The authors find (perhaps not surprisingly) that dsDNA is preferred and in fact this substrate can get fully integrated. Solid ligation is also observed for ssDNA while ssRNA is only weakly ligated and the authors speculate that full integration of these substrates might require additional, unknown, host factors. However, the rationale behind the ligation experiment is not clear to me, because while the use of RNA for spacer acquisition is an intriguing idea, surely this would have to be reverse transcribed into DNA before integration? Therefore, I am not sure what the significance of the observed ssRNA (or ssDNA, for that matter) ligation is. This is an important point that the authors should address in their revision.

Moreover, if the addition of dNTPs in the integration experiments with ssDNA and ssRNA as substrates is supposed to support the transcription activity of the RT domain, would it make sense to include a primer to support this activity, as is done for the RT activity measurement?

Another major question relates to the lack of DNA in the structure. The authors prepare a complicated DNA substrate that as far as I understand consists of four different oligos that must anneal correctly in order to create the branched, ligated half site intermediate. This is then mixed in a 1:4 molar ratio with the Cas1 hybrid/Cas2 (12.5 mM to 50 mM each) and it is concluded that protein and DNA co-migrate during gel filtration (Supplementary Figure 1A). However, there is very little data to support this conclusion so I can only assume that this is based on the app. 1:1 ratio of OD280 to 260 observed during gel filtration? But if the DNA wasn't added in molar excess (1 DNA substrate is added per 4 Cas1/Cas complexes) what does the large DNA peak during gel filtration then represent? I simply cannot reconcile this gel filtration trace with the conclusion that there is full binding and given the results from cryo EM, I strongly suspect that they do in fact not bind each other. This is perhaps not such a big issue as the structure is basically assumed to be DNA-less, however, it is very confusing to the reader. So, I think it is essential that the authors work out whether DNA is bound or not, and adjust the manuscript accordingly. First, I would re-run the gel filtration column with protein only, DNA only and protein+DNA always in the same quantities and compare. If this indeed shows binding, the manuscript can be adjusted to say that DNA is somehow lost during EM grid preparation (air-water interface?) or is too flexible to see. If not, the conclusion that DNA binds should be modified. Also, if DNA is bound, I suggest that an EMSA experiment where one strand is labelled and the other 3 strands are unlabelled and added one after another should be included to demonstrate that the substrate really forms correctly and contains all four pieces.

The Cas6-RT-Cas1 cryo-EM structure lacks two Cas6-RT domains out of four, as mentioned in the text, because of the flexibility of Cas6-RT domains. This explanation is totally plausible. However, it would be helpful to see an SDS-PAGE (maybe in Supplementary Material) showing that the purified protein is full length and does not undergo the proteolytic cleavage during the purification process.

The argument on page 7 relating to the "uncharacterised density" in one Cas1 subunit needs some work. First of all, it's nearly impossible to see this density in the two figures where it is shown. I would like to see a close-up view with all (i.e. non-carved) density and nearby residues shown as sticks to assess what it might be. The authors suggest that this might be a water molecule or an ion, but can one really expect to see such details at 3.7 Å? Depends on the local resolution, I guess, which again can only be judged if more density is shown. Could it be a partially occupied RT helix given the flexibility of the latter?

Supplementary Table 3 lists two structures, a partial and a complete, but it is not clear from the text what these represent and to what extent they differ. This should be better explained in the main text.

Finally, I am concerned about the degradation products in Supplementary Figure 5 and 6. These appear to occur primarily when wt Cas1 fusion is present, and less with the H873A mutant. But are they due to the activity of Cas1 or could there be co-purified nucleases in the wt prep?

Minor suggestions:
Page 5. Do you want to comment on why Cas2 was built de novo given that high res crystal structures of homologous enzymes are available (from your own lab even)? Are they too different to be useful?

Page 6. Please show the homology between the RT fingers domains (and the lack of homology between palm domains) in a figure (or refer to Figure 5b if this covers it).

Page 6. Last sentence starting "Previous biochemical and genomic..." is lacking a reference.

Page 7. Bottom. How do you know that the Cas1 subunit that contains a helix from the RT in its active site is "inactive"? Blocked, perhaps?

Page 8. The argument that the "blue" sequence motif is characteristic of RT-linked Cas1 proteins is not strong. The two logos are very similar. Perhaps the His in the last position is significant, so consider revising the argument.

Page 8. Bottom. Please state (either in the text or figure) the expected length of the ligated products. Is it 49+35+15? It's relevant when trying to understand what the 120-130 bp band is in Supplementary Figure 5. Also, 5b and 5c are denaturing gels, right? In this case it is somewhat misleading to show non-covalently coupled duplexes on the right-hand side.

Methods. Please state incubation temperature for complex formation for cryo-EM.

Methods. EMPIAR entry code missing in methods and Supplementary Table 3.

Figure 2. Legend. "Open-circle (OC)" should be "Open circular (OC)".

Figure 5e. It confused me in the beginning that the DNA/RNA sequences are so long that they extend into the neighbouring parts of the gel. Perhaps show them on the side?

Suppl. Figure 1A. Consider including an SDS-PAGE with the final purified complex, and column total and void volumes as well as elution volumes of standard proteins should be indicated on the chromatogram.

Suppl. Figure 2a. The micrograph has some weird squares when zoomed. This could simply be a jpg compression artefact, but please check. Also, I suggest you circle particles that were picked.

Suppl. Figure 5. As stated above, it needs to be much clearer, which covalently linked species you think migrate where and what their expected lengths are.

Suppl. Figure 7c. Why is so much RNA required to see the reaction? And why are the dsDNA and ssDNA products so weak? Compared to Figure 2b, that is.

Suppl. Table 3. EMPIAR entry code missing. What are the two structures? This should be mentioned in the main text.

Reviewer #2 (Remarks to the Author):

In this manuscript Wang et al provide a structure function analysis of a CRISPR reverse transcriptase-integrase complex (Cas6-RT-Cas1:Cas2). This complex catalyzes the integration of new spacers (adaptation) from RNA spacers and, via Cas6, catalyzes the processing of premature crRNA's. There is growing interest in adaptation-based tools and in particular the RT fused systems as these can be used to record cellular transcriptional activity.

First, the authors present the cryo-EM structure of the Cas6-RT-Cas1:Cas2 complex to an overall resolution of 3.7A, with core regions at 3.3A resolution (Figure 1). The Cas1:Cas2 is arranged similarly to previous Cas1:Cas2 structures with two dimers of Cas1 bridged by a dimer of Cas2 in a hetero-hexameric complex. Two of the four Cas6-RT moieties are disordered, presumably because of their flexibility. The other two copies are located orthogonal to Cas1 with the RT domain packed against the Cas2 dimer and Cas6 packed against the RT domain. Analysis of the structure of the RT domain suggests that the observed conformation maybe inactive (Figure 5).

Next, the authors tested the substrate preferences of the complex in in vitro cleavage-ligation reactions finding that DNA substrates are integrated much more efficiently than RNA substrates (Figure 2). Integration of RNA substrates is unique to RT fused Cas1's. Using a mutagenesis strategy, guided by the cryo-EM structure, the authors identify R835 as an important element for RNA integration, which is absent in stand-alone Cas1's (Figure 2). They also found that integration of ssDNA and dsDNA substrates has a length dependence. Although this was expected for the dsDNA substrate it is surprising for the ssDNA as in this case length dependence would not depend on interaction with a DNA duplex or access to two 3'-OH ends (Figure 3).

The authors also determined whether the Cas6-RT-Cas1 complex can catalyze full-site integration using an in vitro reaction into a selectable plasmid system. They found that 14 out of 25 insertion events generated full-sites, 12 of these located at the leader-repeat junction. The other events resulting in incomplete or abortive integration and occurred at off-target sites (Figure 4).

Using end labeled substrates the authors show that the Cas6-RT-Cas1:Cas2 complex is able to catalyze DNA primer extension with dNTPs using DNA and RNA templates with slightly higher fidelity for RNA than DNA (Figure 5).

Finally, combining their biochemical assays and structural analysis the authors probed the crosstalk between Cas6, RT and Cas1 active sites. These studies revealed a bidirectional cross-talk between the Cas1 and RT domains that is, at least in part, mediated by a RT-helix that connects the RT ad Cas1 active sites. They also revealed unidirectional crosstalk between Cas6 and the Cas1 and RT active sites, with Cas6 mutants significantly affecting Cas1 and RT activities but Cas1 and RT mutations having no effect on Cas6 RNA processing.

This is a well written and executed study, whose conclusions are justified by the results. This is a very interesting system that is shrouded in mystery with regards to its mechanism. This paper provides the first high-resolution picture of the cas6-RT-Cas1:Cas2 complex, which will serve as a blueprint for dissecting its mechanisms. Indeed, follow-up biochemical experiments presented here provide new insight into the coordination of the three active sites of this complex. This work will be of interest to the broad readership of Nature Communications.

RESPONSE TO REVIEWER COMMENTS

Reviewer #1 (Remarks to the Author):

Wang et al. present structural and functional studies of an unusual CRISPR-Cas integration complex consisting of Cas2 and a Cas1 protein, which is naturally linked to reverse transcriptase and Cas6 enzymes. The structure reveals an unusual higher-order structure in which Cas1 and Cas2 interact in a non-canonical way and the active sites of two (out of four) Cas1 subunits are blocked by a helix from the RT enzyme. Functional studies reveal that while the enzyme can ligate both ssDNA, dsDNA, and ssRNA substrates, only dsDNA can get fully integrated in vitro and in vivo.

This manuscript presents novel and highly interesting insights into an unusual CRISPR-Cas system, which strongly complements and extends earlier studies of RT-containing integration systems. The structural work is sound and the conclusions drawn from this are mostly warranted given the modest resolution (3.7 Å). The text is illustrated by beautiful figures that are mostly very clear. Nevertheless, I find that some of the biochemistry and functional studies require additional support to complete the story.

A major outstanding question is how the RT domain works in connection with the integration functions as classically supported by Cas1/Cas2. The functional studies address this in several ways, initially through analysis of ligation of ssDNA, ssRNA, and dsDNA into a plasmid-borne CRISPR array. The authors find (perhaps not surprisingly) that dsDNA is preferred and in fact this substrate can get fully integrated. Solid ligation is also observed for ssDNA while ssRNA is only weakly ligated and the authors speculate that full integration of these substrates might require additional, unknown, host factors. However, the rationale behind the ligation experiment is not clear to me, because while the use of RNA for spacer acquisition is an intriguing idea, surely this would have to be reverse transcribed into DNA before integration? Therefore, I am not sure what the significance of the observed ssRNA (or ssDNA, for that matter) ligation is. This is an important point that the authors should address in their revision.

Response: While dsDNA substrates have been shown to be the preferred substrate for CRISPR spacer integration in classical Cas1/Cas2 systems, it is unclear what the substrate preferences might be for CRISPR systems containing an RT domain. Previous studies examining RNA-derived spacer acquisition in RT-associated CRISPR systems suggested that RNA might be a major or even preferred substrate in these systems. Based on those observations, we wanted to test whether our system might be capable of conducting expanded rather than

specialized acquisition of sequences from RNA and DNA and from single stranded and double-stranded substrates.

To do so, we conducted experiments to examine the Cas6-RT-Cas1—Cas2 complex's integrase activities in the context of half-site integration reactions (the first step of integration: protospacer ligation into the CRISPR array) and full-site integration reactions (completed integration). The rationale behind the ligation experiment is to examine the complex's ability to conduct half-site integration reactions with these different substrates. Although we did not observe full-site integration with ssDNA and ssRNA substrates, we believe that the results for the half-site integration reactions are still significant and informative, because they suggest that the complex is able to accept these substrates for half-site integrations, a characteristic not observed in many classical Cas1/Cas2 systems.

In response to the point ***"surely this [RNA protospacer] would have to be reverse transcribed into DNA before integration?"*****, it is not yet certain whether the RNA substrate gets reverse transcribed before or after the half-site integration reaction, based on our results and previous studies' observations. We believe it is important to test the ssRNA and ssDNA ligation, because the ability for the complex to catalyze ligation of a ssRNA or ssDNA substrate into the CRISPR array opens up the possibility for the ligated single-stranded substrate to get reverse transcribed (or transcribed in the case of ssDNA) after the half-site integration reaction, as suggested by Silas et al. (2016).**

Moreover, if the addition of dNTPs in the integration experiments with ssDNA and ssRNA as substrates is supposed to support the transcription activity of the RT domain, would it make sense to include a primer to support this activity, as is done for the RT activity measurement?

Response: We have performed ligation reactions with these substrates in the presence or absence of dNTPs, and as shown in Supplementary Figure 6, ligation occurs whether or not dNTPs are supplied. We refer to this in the text on p. 9: "Furthermore, unlike what is observed for the MMB-1 system, dNTPs are not required for RNA ligation."

Although the complex is able to ligate RNA substrates in the absence of dNTPs, we have taken the reviewer's suggestion to test a DNA primer / RNA template substrate, as done for the RT activity measurement, in a cleavage-ligation experiment. In this experiment, we tested the plasmid-nicking activity in the presence of Substrate A (the substrate used for the RT activity assays) and

Substrate B (the reverse transcription product of Substrate A) with and without dNTPs. We performed these experiments in triplicate and observed similar plasmid-nicking levels with both substrates (89% and 91% for Substrate A and Substrate B, respectively, without dNTPs after 2 hours and 95% and 97% for Substrate A and Substrate B, respectively, with dNTPs after 2 hours). These data also indicate that dNTPs are not required for cleavage-ligation activity. We have included these new data in Supplementary Figure 5b and address this in the text.

Another major question relates to the lack of DNA in the structure. The authors prepare a complicated DNA substrate that as far as I understand consists of four different oligos that must anneal correctly in order to create the branched, ligated half site intermediate. This is then mixed in a 1:4 molar ratio with the Cas1 hybrid/Cas2 (12.5 mM to 50 mM each) and it is concluded that protein and DNA co-migrate during gel filtration (Supplementary Figure 1A). However, there is very little data to support this conclusion so I can only assume that this is based on the app. 1:1 ratio of OD280 to 260 observed during gel filtration? But if the DNA wasn't added in molar excess (1 DNA substrate is added per 4 Cas1/Cas complexes) what does the large DNA peak during gel filtration then represent? I simply cannot reconcile this gel filtration trace with the conclusion that there is full binding and given the results from cryo EM, I strongly suspect that they do in fact not bind each other.
This is perhaps not such a big issue as the structure is basically assumed to be DNA-less, however, it is very confusing to the reader. So, I think it is essential that the authors work out whether DNA is bound or not, and adjust the manuscript accordingly. First, I would re-run the gel filtration column with protein only, DNA only and protein+DNA always in the same quantities and compare. If this indeed shows binding, the manuscript can be adjusted to say that DNA is somehow lost during EM grid preparation (air-water interface?) or is too flexible to see. If not, the conclusion that DNA binds should be modified. Also, if DNA is bound, I suggest that an EMSA experiment where one strand is labelled and the other 3 strands are unlabelled and added one after another should be included to demonstrate that the substrate really forms correctly and contains all four pieces.

Response: We appreciate these suggestions and have carried out the suggested experiments. We re-ran the gel filtration experiments with protein only, DNA only, and protein + DNA in the same quantities. Although the gel filtration curves show that most of the DNA (which was provided in stoichiometric excess) is unbound, the OD260:OD280 ratio changes from 0.55 for the apo Cas6-RT-Cas1—Cas2 peak to 0.93 for the Cas6-RT-Cas1—Cas2 + DNA peak. The retention volume for this peak also changes from 12.1 mL for the apo Cas6-RT-Cas1—Cas2 peak to 11.6 mL for the Cas6-RT-Cas1—Cas2 + DNA peak. When we analyzed a sample from the

Cas6-RT-Cas1—Cas2 + DNA peak at 11.6 mL (the same sample collected for cryoEM analysis) on an SDS-PAGE gel and a denaturing urea-PAGE gel (post-stained with SYBR Gold), we observed species corresponding to all expected protein and nucleic acid components of the half-site DNA substrate-bound Cas6-RT-Cas1-Cas2 complex.

We also took the reviewer's suggestion to run EMSA experiments with half-site substrates where only one strand is fluorescently labeled. We performed this with 4 substrate variations, each with a different strand labeled. To form each substrate, we annealed the four strands to form the substrate and then purified the correct substrate on a native PAGE gel as done for the unlabeled half-site substrate in the gel filtration experiments. We have checked these substrates on a denaturing gel post-stained with SYBR Gold to ensure that the substrate has formed correctly and all four strands are present. The EMSA experiments support the conclusion that the DNA substrate binds the protein complex. We have included the new data for the gel filtration traces and EMSA experiments in Supplementary Figure 1. Together, these data show that the sample collected for cryoEM is the DNA-bound protein complex, and we conclude that the DNA was lost during EM grid preparation or is too flexible to see.

The Cas6-RT-Cas1 cryo-EM structure lacks two Cas6-RT domains out of four, as mentioned in the text, because of the flexibility of Cas6-RT domains. This explanation is totally plausible. However, it would be helpful to see an SDS-PAGE (maybe in Supplementary Material) showing that the purified protein is full length and does not undergo the proteolytic cleavage during the purification process.

Response: As suggested, we have included an SDS-PAGE gel image in Supplementary Figure 1 showing that the purified protein is full length.

The argument on page 7 relating to the "uncharacterised density" in one Cas1 subunit needs some work. First of all, it's nearly impossible to see this density in the two figures where it is shown. I would like to see a close-up view with all (i.e. non-carved) density and nearby residues shown as sticks to assess what it might be. The authors suggest that this might be a water molecule or an ion, but can one really expect to see such details at 3.7 Å? Depends on the local resolution, I guess, which again can only be judged if more density is shown. Could it be a partially occupied RT helix given the flexibility of the latter?

Response: We have improved this figure as suggested by including a close-up view with all density and nearby residues shown as sticks. The unaccounted-for

density remains spherical even at low thresholds and does not resemble the RT-helix density.

Supplementary Table 3 lists two structures, a partial and a complete, but it is not clear from the text what these represent and to what extent they differ. This should be better explained in the main text.

Response: We have clarified the names used to refer to each of these structures and added language to further clarify their differences in the main text.

Finally, I am concerned about the degradation products in Supplementary Figure 5 and 6. These appear to occur primarily when wt Cas1 fusion is present, and less with the H873A mutant. But are they due to the activity of Cas1 or could there be co-purified nucleases in the wt prep?

Response: To address the concern of co-purified nucleases in the WT prep, we have included SDS-PAGE gels of the purified WT Cas1 fusion prep and the H873A mutant prep in Supplementary Figure 6b. The similar purity of the two preps suggest that the degradation products are likely due to Cas1 activity and not due to co-purified nucleases.

Minor suggestions:
Page 5. Do you want to comment on why Cas2 was built de novo given that high res crystal structures of homologous enzymes are available (from your own lab even)? Are they too different to be useful?

Response: The present Cas2 structure was sufficiently different from previous structures and local resolution was high enough for de novo modeling.

Page 6. Please show the homology between the RT fingers domains (and the lack of homology between palm domains) in a figure (or refer to Figure 5b if this covers it).

Response: We have added an additional panel to Supplementary Figure 4 to better highlight the similarities and differences between these two RT structures.

Page 6. Last sentence starting "Previous biochemical and genomic..." is lacking a reference.

Response: We have included references to the last sentence.

Page 7. Bottom. How do you know that the Cas1 subunit that contains a helix from the RT in its active site is "inactive"? Blocked, perhaps?

Response: At the bottom of page 7, the “inactive” Cas1b domain that we are referring to is the subunit that is most likely the non-catalytic Cas1 subunit based on what is known of the equivalent Cas1 subunit in classical Cas1-Cas2 systems (it plays a structural role and is not involved in catalyzing integration). We have changed the term “inactive” to “non-catalytic.” As we have explained in the text, this subunit is actually not the one that contains a helix from the RT in its active site, and it is the catalytic Cas1a domain that accommodates this insertion.

Page 8. The argument that the "blue" sequence motif is characteristic of RT-linked Cas1 proteins is not strong. The two logos are very similar. Perhaps the His in the last position is significant, so consider revising the argument.

Response: We have revised this section to clarify that our decision to explore the potential role of residue R835 in protospacer ligation is based primarily on our structural observations. We acknowledge that the two logos are similar (though these residues are not conserved in the E. Coli Cas1, which has been the most commonly used system to study classical Cas1-Cas2 systems thus far).

Page 8. Bottom. Please state (either in the text or figure) the expected length of the ligated products. Is it 49+35+15? It's relevant when trying to understand what the 120-130 bp band is in Supplementary Figure 5. Also, 5b and 5c are denaturing gels, right? In this case it is somewhat misleading to show non-covalently coupled duplexes on the right-hand side.

Response: We appreciate this suggestion. We have made the suggested changes to include the expected lengths of the ligated products and to clarify the expected products on the denaturing gels.

Methods. Please state incubation temperature for complex formation for cryo-EM.
Methods. EMPIAR entry code missing in methods and Supplementary Table 3.

Response: We have included the incubation temperature for complex formation in the methods and the EMPIAR accession ID in the methods and Supplementary Table 3.

Figure 2. Legend. "Open-circle (OC)" should be "Open circular (OC)".

Response: We have made the according changes from “open-circle” to “open circular”.

Figure 5e. It confused me in the beginning that the DNA/RNA sequences are so long that they extend into the neighbouring parts of the gel. Perhaps show them on the side?

Response: We have adjusted the figure to address this concern.

Suppl. Figure 1A. Consider including an SDS-PAGE with the final purified complex, and column total and void volumes as well as elution volumes of standard proteins should be indicated on the chromatogram.

Response: Thank you for this suggestion. We have included an SDS-PAGE with the final purified complex and the above suggested information in Supplementary Figure 1.

Suppl. Figure 2a. The micrograph has some weird squares when zoomed. This could simply be a jpg compression artefact, but please check. Also, I suggest you circle particles that were picked.

Response: We have re-exported the micrograph as a png with minimal compression to avoid these artifacts and circled particles which contributed to the final reconstruction.

Suppl. Figure 5. As stated above, it needs to be much clearer, which covalently linked species you think migrate where and what their expected lengths are.

Response: We have made the according changes to clarify the covalently linked species and what their expected lengths are.

Suppl. Figure 7c. Why is so much RNA required to see the reaction? And why are the dsDNA and ssDNA products so weak? Compared to Figure 2b, that is.

Response: To address the first question, we observe much lower efficiencies with RNA substrates compared to DNA substrates for cleavage-ligation and we address this in the main text. We believe that these differences in efficiency may suggest that RNA may not be the primary source of spacers for this system or that additional host factors are required to improve the efficiency of RNA integration. We also consider the possibility that the efficiency in integrating RNA

substrates varies depending on the substrate as a part of a selection process to distinguish between self and non-self.

To address the second question, the initial fluorescent gel comparing the three substrates in Figure 2b as well as the gel for the ssRNA ligation experiment in Supplementary Figure 7c (now Supplementary Figure 8c) were imaged using a PMT voltage of 750 V. The fluorescent gels for the dsDNA and ssDNA ligation experiments in Supplementary Figure 7c (now Supplementary Figure 8c) were imaged using a lower PMT voltage of 650 V to ensure that none of the product bands were saturated, since the percent ligation activity relative to WT is calculated based on the fraction of fluorescent products from the mutant complex relative to that from the WT complex.

Suppl. Table 3. EMPIAR entry code missing. What are the two structures? This should be mentioned in the main text.

Response: We have updated the table to include the EMPIAR accession code, clarified the names used to refer to each of these structures and added language to further clarify their differences in the main text.

Reviewer #2 (Remarks to the Author):

In this manuscript Wang et al provide a structure function analysis of a CRISPR reverse transcriptase-integrase complex (Cas6-RT-Cas1:Cas2). This complex catalyzes the integration of new spacers (adaptation) from RNA spacers and, via Cas6, catalyzes the processing of premature crRNA's. There is growing interest in adaptation-based tools and in particular the RT fused systems as these can be used to record cellular transcriptional activity.

First, the authors present the cryo-EM structure of the Cas6-RT-Cas1:Cas2 complex to an overall resolution of 3.7A, with core regions at 3.3A resolution (Figure 1). The Cas1:Cas2 is arranged similarly to previous Cas1:Cas2 structures with two dimers of Cas1 bridged by a dimer of Cas2 in a hetero-hexameric complex. Two of the four Cas6-RT moieties are disordered, presumably because of their flexibility. The other two copies are located orthogonal to Cas1 with the RT domain packed against the Cas2 dimer and Cas6 packed against the RT domain. Analysis of the structure of the RT domain suggests that the observed conformation maybe inactive (Figure 5).

Next, the authors tested the substrate preferences of the complex in in vitro cleavage-ligation reactions finding that DNA substrates are integrated much more efficiently than RNA substrates (Figure 2). Integration of RNA substrates is unique to RT fused Cas1's. Using a mutagenesis strategy, guided by the cryo-EM structure, the authors identify R835 as an important element for RNA integration, which is absent in stand-alone Cas1's (Figure 2). They also found that integration of ssDNA and dsDNA substrates has a length dependence. Although this was expected for the dsDNA substrate it is surprising for the ssDNA as in this case length dependence would not depend on interaction with a DNA duplex or access to two 3'-OH ends (Figure 3).

The authors also determined whether the Cas6-RT-Cas1 complex can catalyze full-site integration using an in vitro reaction into a selectable plasmid system. They found that 14 out of 25 insertion events generated full-sites, 12 of these located at the leader-repeat junction. The other events resulting in incomplete or abortive integration and occurred at off-target sites (Figure 4).

Using end labeled substrates the authors show that the Cas6-RT-Cas1:Cas2 complex is able to catalyze DNA primer extension with dNTPs using DNA and RNA templates with slightly higher fidelity for RNA than DNA (Figure 5).

Finally, combining their biochemical assays and structural analysis the authors probed the crosstalk between Cas6, RT and Cas1 active sites. These studies revealed a bidirectional cross-talk between the Cas1 and RT domains that is, at least in part, mediated by a RT-helix that connects the RT ad Cas1 active sites. They also revealed unidirectional crosstalk between Cas6 and the Cas1 and RT active sites, with Cas6 mutants significantly affecting Cas1 and RT activities but Cas1 and RT mutations having no effect on Cas6 RNA processing.

This is a well written and executed study, whose conclusions are justified by the results. This is a very interesting system that is shrouded in mystery with regards to its mechanism. This paper provides the first high-resolution picture of the cas6-RT-Cas1:Cas2 complex, which will serve as a blueprint for dissecting its mechanisms. Indeed, follow-up biochemical experiments presented here provide new insight into the coordination of the three active sites of this complex. This work will be of interest to the broad readership of Nature Communications.

Response: We thank Reviewer #2 for their supportive comments on the manuscript.

REVIEWER COMMENTS

Reviewer #1 (Remarks to the Author):

I would like to thank the authors for so carefully considering and handling all my concerns as well as suggestions for improvement. Since the authors have addressed all requested changes, I have no further comments and fully support publication at this stage.

Reviewer #2 (Remarks to the Author):

The authors were very receptive to the reviewers comments. I recommend publication.

Reviewer #3 (Remarks to the Author):

In their manuscript, Wang et al. show the first structure of a complete Cas6-RT-Cas1-Cas2 complex. The structure reveals various remarkable differences to known Cas1-Cas2 complexes, RT domains, and Cas6 proteins, as nicely described by the authors. The authors use a variety of biochemical experiments to demonstrate that the complex can form half-site integration intermediates with dsDNA, ssDNA, and ssRNA as a substrate in vitro. Based on the structure and multiple sequence alignments, a novel strictly conserved residue R834 in the Cas1 domain of RT-Cas1s is identified, and it is demonstrated that this residue is essential for half-site ssRNA integration and simulates DNA integration. Full-site integration of spacers is tested using a previously developed strategy, in which the authors are able to demonstrate full-site integration of dsDNA substrates, but not of ssDNA or ssRNA substrates. This leaves the question if ssDNA or ssRNA are valid substrates in vivo open. Based on the positioning of the domains, the authors form an interesting theory about the regulation of the various activities that the complex can confer. This is subsequently tested and confirmed using an array of catalytic mutants.

The article is generally well written and the experiments support the conclusions. There are numerous new insights into this mysterious system and its mechanism. However, also various 'loose ends' exist. Still, I think that tying these loose ends goes beyond the scope of this article, and instead that they should be seen as an interesting source for future work. In its current state, it is definitely an interesting read for a specialized audience, but I would like to suggest mostly textual changes to help non-expert readers to understand the significance of this work. Furthermore, I have some minor comments, most of which (I think) can be addressed textually.

With kind regards,
Daan Swarts

Minor comments
-The authors describe that they observe interactions between the complex and DNA during SEC, suggesting it is quite tightly bound. Is it possible that the DNA is only present in part of the complexes, and could that explain the observed co-purification/shifts, but the absence of density in the EM maps?

-In Supp. Figure 6B, the authors have investigated integration of substrates into a short dsDNA molecule. Could the authors indicate what they mean with off-target ligation products? Do I interpret from the gel correctly that substrates are ligated together? How is that possible with a 5' fluorophore labelled substrate? I would expect that that end is not available for ligation.

-For the experiment in Supp. Figure 6, the authors describe in the manuscript that they have performed these experiments to determine if these reactions are site-specific. It's not entirely clear

what is meant with that, as the integration site (sequence?) does not seem to be varied in this experiment. Furthermore, it's not entirely clear what can be concluded from the experiment displayed in Supp. Figure 6C. I am assuming this is a denaturing urea PAGE in which this 15nt stretch is nicked and unwound from the dsDNA target for integration? If so, this should be made clear in at least the figure text.

-I think the conclusion that impairing RT activity or RT-helix-Cas1a interaction critically disrupts RNA ligation is too strong given that RNA ligation activity is still observed (although this cannot be observed from the figures, it is stated in the manuscript text). This should be rephrased.

-The authors regularly use the word significant in their manuscript, but I don't see any statistical analysis performed (other than calculation of error bars). Based on error bars I would also guess that indeed probably the observed effects are significant, statistical tests should be performed if the term significant is to be used.

-In both the figures and in the manuscript text, it is often not directly clear what protein (domain) is meant when referring to substitution mutants. This could easily be avoided by - for example – describing a mutant as Cas6R37A instead of just R37A. I think this would help in getting the point across that mutants in one protein will affect the activity of another protein (or protein domain).

-In the discussion, it is stated that it is unclear when the RNA substrate is reverse transcribed into cDNA during spacer acquisition. Is it strictly necessary for the system to be able to do this? While RNA substrates appear to be integrated (at low efficiency), could it be that the RT domain is only functioning as a DNA polymerase? In Figure 5e, it appears that RT activity is extremely low compared to DNA polymerase activity shown in the same figure.

-Some of the fitting to the electron density is extremely poor (e.g. Chain E) as can be observed in the validation report. Can the authors justify building these residues?

Textual suggestions

Page 3:
-I'm not sure if immunity can 'begin'. Suggested to change to 'immunity is obtained'.
-Suggested change: ... that assemble with effector complexes > that assembly with Cas proteins to form effector complexes that identify...
-Suggested change: ...derived from DNA phage > derived from phage DNA

Page 4:
-Maybe it can be explained for the non-expert audience why it is surprising that the RT-integrase favour DNA as an integration substrate.

Page 6:
-Fewer B-strands… > suggested to say exactly how much

Page 7:
-Suggested change: or single-stranded RNA (ssRNA) substrate > or a single-stranded RNA (ssRNA) substrate

-a supercoiled target plasmid ◊ the term target might be confusing in the context of CRISPR-Cas systems. I would suggest to change it to: a supercoiled plasmid that functions as target for spacer integration.

-Suggested change: …fluorophore into the target DNA molecule > fluorophore-labelled substrate into the CRISPR locus in the target plasmid.

Page 8

--Suggested change: may plan an important role for the Cas6-RT-Cas1's function > may plan an important role for the function of Cas6-RT-Cas1

-The authors have only investigated the role of R835 (and H873), not all residues that were identified on this page. However, this is suggested in the first sentence 'To examine the potential role of these residues in protospacer ligation'. I suggest rephrasing to make this clear.

Page 9

-Also here I would make clear that the linear target DNA is the target for space integration: e.g. using a short dsDNA comprising…. as target for integration of the substrate.

Figures:

-In Figure 2, panel b, it is unclear what proteins are present (i.e. the figure suggests only Cas1 and/or Cas2, while I think its Cas6-RT-Cas1 and/or Cas2?).

-In Supplemental Figure 6A, it would be helpful if the fluorophores are indicated like in panel B. In panel B, it is unclear what is exactly in each lane (in 6C, it is clear).

RESPONSE TO REVIEWER COMMENTS

Reviewer #1 (Remarks to the Author):

I would like to thank the authors for so carefully considering and handling all my concerns as well as suggestions for improvement. Since the authors have addressed all requested changes, I have no further comments and fully support publication at this stage.

Response: We thank Reviewer #1 for their supportive comments on the manuscript.

Reviewer #2 (Remarks to the Author):

The authors were very receptive to the reviewers comments. I recommend publication.

Response: We thank Reviewer #2 for their supportive comments on the manuscript.

Reviewer #3 (Remarks to the Author):

In their manuscript, Wang et al. show the first structure of a complete Cas6-RT-Cas1-Cas2 complex. The structure reveals various remarkable differences to known Cas1-Cas2 complexes, RT domains, and Cas6 proteins, as nicely described by the authors. The authors use a variety of biochemical experiments to demonstrate that the complex can form half-site integration intermediates with dsDNA, ssDNA, and ssRNA as a substrate in vitro. Based on the structure and multiple sequence alignments, a novel strictly conserved residue R834 in the Cas1 domain of RT-Cas1s is identified, and it is demonstrated that this residue is essential for half-site ssRNA integration and simulates DNA integration. Full-site integration of spacers is tested using a previously developed strategy, in which the authors are able to demonstrate full-site integration of dsDNA substrates, but not of ssDNA or ssRNA substrates. This leaves the question if ssDNA or ssRNA are valid substrates in vivo open. Based on the positioning of the domains, the authors form an interesting theory about the regulation of the various activities that the complex can confer. This is subsequently tested and confirmed using an array of catalytic mutants.

The article is generally well written and the experiments support the conclusions. There are numerous new insights into this mysterious system and its mechanism. However, also various 'loose ends' exist. Still, I think that tying these loose ends goes beyond the scope of this article, and instead that they should be seen as an interesting source for future work. In its current state, it is definitely an interesting read for a specialized audience, but I would like to suggest mostly textual changes to help non-expert readers to understand the significance of this work. Furthermore, I have some minor comments, most of which (I think) can be addressed textually.

With kind regards,
Daan Swarts

Minor comments
-The authors describe that they observe interactions between the complex and DNA during SEC, suggesting it is quite tightly bound. Is it possible that the DNA is only present in part of the complexes, and could that explain the observed co-purification/shifts, but the absence of density in the EM maps?

Response: We thank Reviewer 3 for their suggestion. While our gel filtration and EMSA data suggest that there is binding between the complex and DNA, we agree that it is possible that the DNA is only present in some of the complexes and could explain the absence of density in the EM maps, although notably, we have not been able to determine the structure from the sample that was collected from the apo complex that was not prepared with the DNA substrate. We have revised the text to include this possibility in addition to the possibility that DNA was lost during grid preparation or is too flexible to see.

-In Supp. Figure 6B, the authors have investigated integration of substrates into a short dsDNA molecule. Could the authors indicate what they mean with off-target ligation products? Do I interpret from the gel correctly that substrates are ligated together? How is that possible with a 5' fluorophore labelled substrate? I would expect that that end is not available for ligation.

Response: The expected on-target ligation product is the 119 nt ligation product from the Cas1-catalyzed cleavage-ligation reaction (resulting from the nucleophilic attack of the protospacer at the end of the repeat - the target integration site - shown in Supp. Figure 6a). What we mean by off-target ligation products are the products resulting from nucleophilic attacks by the protospacer that do not occur at the ends of the repeat (and instead happen in the middle of the leader, repeat, or spacer sequence). These Cas1 cleavage-ligation reactions do not require free 5' ends in the substrate. We have adjusted the text to make this clear.

-For the experiment in Supp. Figure 6, the authors describe in the manuscript that they have performed these experiments to determine if these reactions are site-specific. It's not entirely clear what is meant with that, as the integration site (sequence?) does not seem to be varied in this experiment. Furthermore, it's not entirely clear what can be concluded from the experiment displayed in Supp. Figure 6C. I am assuming this is a denaturing urea PAGE in which this 15nt stretch is nicked and unwound from the dsDNA target for integration? If so, this should be made clear in at least the figure text.

Response: We thank the reviewer for their comments. To clarify, the experiments in Supp. Figure 6 were conducted to examine the site-specificity of the integrase for the ends of

the repeat sequence. This is an important characteristic of the integrase that preserves genome integrity, since off-target integration could be toxic. In this case, we examined whether or not the integrase prefers to conduct cleavage-ligation with the protospacer at the ends of the repeat (as opposed to the middle of the leader, repeat, or spacer sequences of the target molecule). The expected reaction occurring at the correct target site is depicted in Supp. Figure 6a. We examine the actual integration / cleavage-ligation sites by labeling either the protospacer (Supp. Figure 6b) or the target dsDNA molecule (Supp. Figure 6c) and analyzing the products on a denaturing urea PAGE gel. Both Supp. Figure 6b+c support the conclusion that the main cleavage-ligation product is the expected product depicted in Supp. Figure 6a (with the nucleophilic attack by the protospacer at the end of the repeat sequence) resulting in a 119-nt strand containing the protospacer and the 15-nt spacer strand. This suggests that the integrase has some target recognition in being able to recognize the ends of the repeat as the target integration site (although this site-specificity is somewhat comprised as shown by the presence of off-target products).

Supp. Figure 6c is additionally informative because it shows that there is no extension of the 15-nt strand following cleavage-ligation when dNTPs are added (unlike what is observed for the MMB-1 system in previous studies that suggest target-primed reverse transcription). We have adjusted the text and figure to make this clear.

-I think the conclusion that impairing RT activity or RT-helix-Cas1a interaction critically disrupts RNA ligation is too strong given that RNA ligation activity is still observed (although this cannot be observed from the figures, it is stated in the manuscript text). This should be rephrased.

Response: We thank the reviewer for their suggestion and have rephrased this in the text.

-The authors regularly use the word significant in their manuscript, but I don't see any statistical analysis performed (other than calculation of error bars). Based on error bars I would also guess that indeed probably the observed effects are significant, statistical tests should be performed if the term significant is to be used.

Response: We thank the reviewer for their suggestion. We have assessed significance using unpaired, two-tailed t tests (alpha = 0.05) and included this in the figure legends and methods section of the text.

-In both the figures and in the manuscript text, it is often not directly clear what protein (domain) is meant when referring to substitution mutants. This could easily be avoided by - for example – describing a mutant as Cas6R37A instead of just R37A. I think this would help in getting the point across that mutants in one protein will affect the activity of another protein (or protein domain).

Response: We thank the reviewer for their suggestion and incorporated these descriptions for the mutants.

-In the discussion, it is stated that it is unclear when the RNA substrate is reverse transcribed into cDNA during spacer acquisition. Is it strictly necessary for the system to be able to do this? While RNA substrates appear to be integrated (at low efficiency), could it be that the RT domain is only functioning as a DNA polymerase? In Figure 5e, it appears that RT activity is extremely low compared to DNA polymerase activity shown in the same figure.

Response: We have adjusted the text to consider the possibility that the RT domain is only functioning as a DNA polymerase. We acknowledge that the DNA polymerase activity may be higher although the RT activity appears to have higher fidelity to the template sequence from the dNTP dropout experiments.

-Some of the fitting to the electron density is extremely poor (e.g. Chain E) as can be observed in the validation report. Can the authors justify building these residues?

We appreciate this concern. In the map of the full complex (EMD-22856), the density for the distal Cas6-RT-Cas1 protomer is weaker, resulting in poorer resolution compared to the rest of the complex. In our model for the full complex (PDB-7KFU) chain E is the Cas6-RT-Cas1 protomer corresponding to that weaker, low-resolution density.

Importantly, chain E in this model (7KFU) was first built in the higher resolution masked partial complex map (EMD-22855) as model (7KFT) and the validation report for that model does not reveal poor fit to the electron density. To build 7KFU, these portions of the refined 7KFT model were then copied and rigid-body docked, and rigid-body refined into the density for the full complex EMD-22856.

We have attempted to make this process and rationale clear to the reader on page 5 in the main text and pages 26-27 in the methods.

Textual suggestions

Page 3:
-I'm not sure if immunity can 'begin'. Suggested to change to 'immunity is obtained'.
-Suggested change: ... that assemble with effector complexes > that assembly with Cas proteins to form effector complexes that identify...
-Suggested change: ...derived from DNA phage > derived from phage DNA

Response: We thank the reviewer for their suggestions and have revised the text.

Page 4:

-Maybe it can be explained for the non-expert audience why it is surprising that the RT-integrase favour DNA as an integration substrate.

Response: We have revised the text to better explain this.

Page 6:
-Fewer B-strands… > suggested to say exactly how much

Response: We have revised the text to include this.

Page 7:
-Suggested change: or single-stranded RNA (ssRNA) substrate > or a single-stranded RNA (ssRNA) substrate

Response: We have incorporated this suggested change.

-a supercoiled target plasmid the term target might be confusing in the context of CRISPR-Cas systems. I would suggest to change it to: a supercoiled plasmid that functions as target for spacer integration.

Response: We thank the reviewer for their suggestion and have incorporated this suggested change.

-Suggested change: …fluorophore into the target DNA molecule > fluorophore-labelled substrate into the CRISPR locus in the target plasmid.

Response: We have incorporated this suggested change.

Page 8
--Suggested change: may plan an important role for the Cas6-RT-Cas1's function > may plan an important role for the function of Cas6-RT-Cas1

Response: We have incorporated this suggested change.

-The authors have only investigated the role of R835 (and H873), not all residues that were identified on this page. However, this is suggested in the first sentence 'To examine the potential role of these residues in protospacer ligation'. I suggest rephrasing to make this clear.

Response: We thank the reviewer for their suggestion and have rephrased this.

Page 9
-Also here I would make clear that the linear target DNA is the target for space integration: e.g. using a short dsDNA comprising…. as target for integration of the substrate.

Response: We thank the reviewer for their suggestion and have rephrased this.

Figures:
-In Figure 2, panel b, it is unclear what proteins are present (i.e. the figure suggests only Cas1 and/or Cas2, while I think its Cas6-RT-Cas1 and/or Cas2?).

Response: We thank the reviewer for their suggestion and have changed the figure to make this clear.

-In Supplemental Figure 6A, it would be helpful if the fluorophores are indicated like in panel B. In panel B, it is unclear what is exactly in each lane (in 6C, it is clear).

Response: We thank the reviewer for their suggestion and have changed the figure to make this clear.
